# Enhancing Photocatalysis: Understanding the Mechanistic Diversity in Photocatalysts Modified with Single‐Atom Catalytic Sites

**DOI:** 10.1002/advs.202303571

**Published:** 2023-10-27

**Authors:** Krzysztof Kruczała, Susann Neubert, Kapil Dhaka, Dariusz Mitoraj, Petra Jánošíková, Christiane Adler, Igor Krivtsov, Julia Patzsch, Jonathan Bloh, Johannes Biskupek, Ute Kaiser, Rosalie K. Hocking, Maytal Caspary Toroker, Radim Beranek

**Affiliations:** ^1^ Faculty of Chemistry Jagiellonian University in Kraków Gronostajowa 2/C1‐21 Krakow 30–387 Poland; ^2^ Faculty of Chemistry and Biochemistry Ruhr University Bochum Universitätsstr. 150 44780 Bochum Germany; ^3^ Department of Materials Science and Engineering Technion – Israel Institute of Technology Haifa 3200003 Israel; ^4^ Institute of Electrochemistry Ulm University Albert‐Einstein‐Allee 47 89069 Ulm Germany; ^5^ Department of Chemical and Environmental Engineering University of Oviedo Oviedo 33006 Spain; ^6^ Chemical Technology Group DECHEMA Research Institute Theodor‐Heuss‐Allee 25 60486 Frankfurt am Main Germany; ^7^ Central Facility of Electron Microscopy Electron Microscopy Group of Material Science University of Ulm D‐89081 Ulm Germany; ^8^ Department of Chemistry and Biotechnology ARC Training Centre for Surface Engineering for Advanced Material SEAM Swinburne University of Technology Hawthorn VIC 3122 Australia; ^9^ The Nancy and Stephen Grand Technion Energy Program Technion – Israel Institute of Technology Haifa 3200003 Israel

**Keywords:** aerobic oxidation, charge separation, electron paramagnetic resonance, oxygen reduction, photocatalysis, single‐atom catalysis

## Abstract

Surface modification of heterogeneous photocatalysts with single‐atom catalysts (SACs) is an attractive approach for achieving enhanced photocatalytic performance. However, there is limited knowledge of the mechanism of photocatalytic enhancement in SAC‐modified photocatalysts, which makes the rational design of high‐performance SAC‐based photocatalysts challenging. Herein, a series of photocatalysts for the aerobic degradation of pollutants based on anatase TiO_2_ modified with various low‐cost, non‐noble SACs (vanadate, Cu, and Fe ions) is reported. The most active SAC‐modified photocatalysts outperform TiO_2_ modified with the corresponding metal oxide nanoparticles and state‐of‐the‐art benchmark photocatalysts such as platinized TiO_2_ and commercial P25 powders. A combination of in situ electron paramagnetic resonance spectroscopy and theoretical calculations reveal that the best‐performing photocatalysts modified with Cu(II) and vanadate SACs exhibit significant differences in the mechanism of activity enhancement, particularly with respect to the rate of oxygen reduction. The superior performance of vanadate SAC‐modified TiO_2_ is found to be related to the shallow character of the SAC‐induced intragap states, which allows for both the effective extraction of photogenerated electrons and fast catalytic turnover in the reduction of dioxygen, which translates directly into diminished recombination. These results provide essential guidelines for developing efficient SAC‐based photocatalysts.

## Graphical abstract

This paper reports novel highly active photocatalysts for the aerobic degradation of pollutants based on anatase TiO_2_ modified with various single‐atom catalysts (SACs), provides valuable insights into the mechanistic diversity of different SACs, and identifies essential design guidelines for the future development of SAC‐based photocatalysts with enhanced performance.

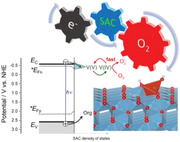

## 1 Introduction

Heterogeneous catalysts with single‐atom (SA) catalytic sites represent a distinct class of catalysts that offer several advantages over conventional metal or metal oxide particle catalysts.^[^ ^1^ ^]^ In addition to improving the atom economy, the active sites of single‐atom catalysts (SACs) possess distinct geometries, electronic structures, and energetics, which can result in superior performance in terms of activity and selectivity. This unique combination of attributes distinguishes SACs from traditional catalysts and highlights their potential for advancing catalytic processes.^[^ ^2^ ^]^ Formation of effective SA catalytic sites also holds great promise in the field of heterogeneous photocatalysis where the deposition of additional catalysts (so called “co‐catalysts”) onto the surface of light‐absorbing photocatalysts is often mandatory for improving the extraction of photogenerated charges (i.e., electrons and holes), thereby enhancing the rate of their reaction with reactants, and thus increasing the quantum yield of photocatalytic processes, which is typically only few percent in the absence of co‐catalysts.^[^ ^3^ ^]^ A particular advantage of SACs in photocatalysis is their very low effective cross section for light absorption compared to that of metal or metal‐oxide catalysts with a fully developed long‐range electronic structure. This makes it easier to avoid parasitic light absorption by the co‐catalyst, which would otherwise block excitation of the light‐absorber and reduce the overall photocatalytic activity.^[^ ^4^ ^]^ Recently, several examples of photocatalysts (e.g., TiO_2_, CdS, BiVO_4_, polymeric carbon nitride, and carbon quantum dots) modified with SACs comprising different metals (e.g., Pt, Ir, Rh, Fe, Co, Ni, Cu, Sn, and In) have been demonstrated for various applications. These applications include the degradation of harmful organic compounds,^[^ ^5^ ^]^ hydrogen evolution,^[^ ^6^ ^]^ oxidation of water to dioxygen,^[^ ^7^ ^]^ useful selective light‐driven conversions^[^ ^8^ ^]^ such as dioxygen reduction to H_2_O_2_,^[^ ^9^ ^]^ selective oxidation of alcohols,^[^ ^10^ ^]^ benzene,^[^ ^11^ ^]^ benzylamine,^[^ ^12^ ^]^ and NO_x_,^[^ ^13^ ^]^ as well as C─C coupling reactions.^[^ ^14^ ^]^ However, our knowledge of the mechanism of photocatalytic enhancement in SAC‐modified photocatalysts remains rather limited,^[^ ^6^, ^15^ ^]^ which makes the rational design of high‐performance photocatalysts based on SACs very challenging.

**Figure 1:**
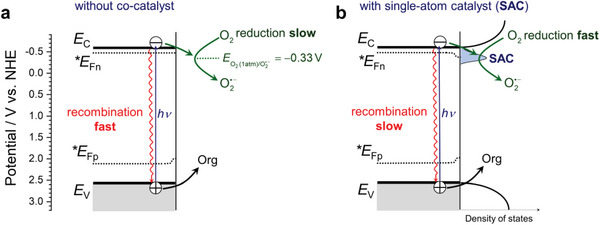
Concept of intensification of photocatalytic degradation of organic pollutant (Org) using single‐atom catalysts (SACs) deposited on TiO_2_ surface. Without any co‐catalyst a) the rate‐limiting reduction of dioxygen by photogenerated electrons is very slow, which renders the electron‐hole recombination very fast. Upon deposition of SACs b), the rate of oxygen reduction is enhanced, which leads to diminished recombination and enhanced degradation rates. *E*_C_, *E*_V_, *E_Fn_, and *E_Fp_ indicate the conduction and valence band edge and the quasi‐Fermi levels (corresponding to the electrochemical potentials, ${}^{*}E_{\mathrm{Fn}}={\tilde{\mu }}_{n}$ and ${}^{*}E_{\mathrm{Fp}}=-{\tilde{\mu }}_{p}$) of electrons and holes, respectively. Note that the higher rate of electron consumption in b) leads to an increased gradient of the quasi‐Fermi level of electrons, which enhances the driving force for charge separation. Ideally, the light absorption cross section of the SAC should be negligible to avoid the blocking of light absorption by TiO_2_, and the electronic states induced by the co‐catalyst should lie energetically close to the quasi‐Fermi level of electrons (*E_Fn_), i.e. just below the conduction band edge of TiO_2_. The band edge positions correspond to pH 7.

In general, the complexity of the various factors influencing the performance of photocatalysts modified with co‐catalysts can be exemplified on TiO_2_‐based photocatalysts for the light‐driven aerobic oxidative degradation of organic pollutants. It has been suggested by Gerischer and Heller already more than thirty years ago^[^ ^3^, ^16^ ^]^ and later confirmed by detailed mechanistic studies,^[^ ^17^ ^]^ that the kinetics of the photocatalytic oxidation of organic molecules on TiO_2_ particles is typically limited by the rate of oxygen reduction by the photogenerated electrons, which is significantly slower (by several orders of magnitude) than the oxidation of organic molecules by the photogenerated holes. Faster consumption of the photogenerated electrons by catalytically enhancing the rate of their reduction reaction with dioxygen is therefore expected to lead to diminished recombination and thus process intensification, as has been demonstrated for TiO_2_ photocatalysts modified with Pt,^[^ ^3^, ^18^ ^]^ CuO_x_, or FeO_x_ nanoparticles as co‐catalysts for oxygen reduction.^[^ ^3^, ^19^ ^]^ Notably, a good co‐catalyst for oxygen reduction should effectively extract electrons from TiO_2_ and enhance catalysis on the surface; however, it should not significantly block light absorption by TiO_2_ and should not induce the formation of energetically deep interfacial trap states that would drastically diminish the reductive power of the trapped electrons and act as recombination centers (**Figure** **1**).

Herein, we report novel SAC‐modified anatase TiO_2_ photocatalysts and demonstrate their superior performance compared to that of pristine TiO_2_ and TiO_2_ modified with metal oxide nanoparticles. With reference to our recent work on rutile TiO_2_ modified with Cu(II) and Fe(III) sites,^[^ ^5c^ ^]^ we extend this concept to anatase TiO_2_ modified with Cu(II) or Fe(III). In addition, we introduce, for the first time, anatase TiO_2_ modified with vanadate SACs and demonstrate its superior photocatalytic performance with respect to benchmark state‐of‐the‐art photocatalysts. Moreover, we demonstrate that the mechanism of activity enhancement of the best‐performing photocatalysts modified with Cu and vanadate SACs differ significantly, particularly with respect to the rate of catalysis of oxygen reduction, which is related to the energetic depth of the SAC‐induced electronic intragap states. Based on the results, specific design rules for developing SAC‐modified photocatalysts with enhanced activity are proposed.

## 2 Results and Discussion

### 2.1 Evidence of Single‐Atom Catalytic Sites

All SAC‐modified photocatalysts were prepared by the impregnation of anatase TiO_2_ powder with very small amounts of copper(II), iron(III) nitrate, or ammonium vanadate. The metal loading was optimized by screening for the highest photocatalytic activity (for details see the Figures S1 and S2, Supporting Information). The best‐performing anatase TiO_2_ photocatalysts comprising Cu(II), Fe(III), or vanadate single atom (SA) catalytic sites are designated as a‐TiO_2_‐Cu**^SA^**, a‐TiO_2_‐Fe**^SA^**, and a‐TiO_2_(400)‐V**^SA^**, having actual metal contents of 0.08 wt.% Cu, 0.11 wt.% Fe, and 0.03 wt.% V, respectively, as determined by inductively coupled plasma optical emission spectrometry (ICP‐OES). Energy dispersive X‐ray spectroscopy (EDX) analysis of the transmission electron microscopy (TEM) data verified the presence of highly distributed Fe, Cu, and V species in very small amounts (order of 0.1 at.%). In the case of vanadium, reliable detection of the element was complicated by the overlap of the dominant Ti K_β_ lines with the V K_α_ lines (Figures S3–S5, Supporting Information). Direct imaging of single Fe(III), Cu(II), and vanadate ions within the TiO_2_ substrate using high‐resolution TEM (HRTEM) or high‐angle annular dark‐field scanning transmission electron microscopy (HAADF‐STEM) was impossible because of the low amounts of Cu, Fe, and V (≈0.1%) and their very low Z‐contrast differences with respect to Ti (Figures S6–S8, Supporting Information). As expected, the presence of Fe(III), Cu(II), and vanadate ions on the surface of TiO_2_ did not lead to any structural changes detectable by X‐ray diffraction; all the samples showed only typical reflections of the anatase TiO_2_ phase (Figure S9, Supporting Information). Raman spectroscopic analysis of a‐TiO_2_(400)‐V**^SA^** was carried out as this sample was heated at 450 °C during the synthesis, which might facilitate structural changes or formation of VO_x_ clusters, but only vibrations related to anatase TiO_2_ were detected (Figure S10, Supporting Information). In contrast, the Raman spectrum of the TiO_2_ sample modified with VO_x_ nanoparticles (a‐TiO_2_(400)‐VOx**^NP^**) revealed additional vibrational contributions ascribed to metavanadate (VO_3_)_n_ (≈940 cm^–1^) and V_2_O_5_ (≈994 cm^–1^).^[^ ^20^ ^]^ All these results are consistent with our assumption that Fe(III), Cu(II), and vanadate ions are present in small amounts as isolated single ions on the surface of the anatase TiO_2_ crystallites.

**Figure 2:**
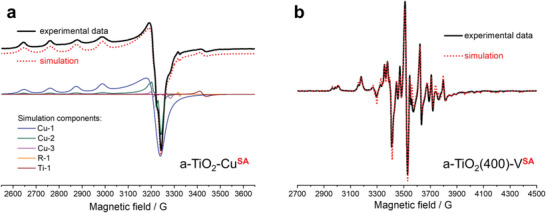
EPR spectroscopic evidence of SAC sites. Experimental (solid black line) and simulated (doted red line) X‐band EPR spectra of a) a‐TiO_2_‐Cu**^SA^** (the simulated components are given at the bottom) recorded in the dark, and b) a‐TiO_2_(400)‐V**^SA^** photocatalysts recorded after 120 min of UV irradiation in the absence of oxygen.

Electron paramagnetic resonance (EPR) spectroscopy is a powerful tool for interrogating systems containing transition metal ions^[^ ^5^, ^21^ ^]^ and can provide direct evidence of the presence of SACs because the spectroscopic fingerprint of isolated metal ions on the surface of TiO_2_ is significantly different from EPR spectra of metal oxide clusters or nanoparticles, which are dominated by long‐range dipolar interactions.^[^ ^5^, ^22^ ^]^ The complex EPR spectrum of a‐TiO_2_‐Cu**^SA^** (**Figure** **2**a) consists of five components, as determined by computer simulations. The magnetic parameters of the main constituent (≈85%), denoted as Cu‐1, are as follows: g_||_ ≅  2.353, A_||_≅ 113 G, and g_⊥_ ≅ 2.067, with an unresolved perpendicular hyperfine structure (HFS) typical of surface‐bound single Cu(II) ions (*d* ^9^ electronic configuration)^[^ ^5^, ^23^ ^]^ or Cu(II) ions substituting lattice Ti(IV),^[^ ^6^, ^24^ ^]^ both expected to be spectroscopically the same. The next two components, Cu‐2 (≈13%, g_||_ ≅  2.354, A_||_≅ 114 G and g_⊥_ ≅ 2.065, A_⊥_≅ 8 G) and Cu‐3 (∼2%, g_||_ ≅  2.353, A_||_≅ 114 G and g_⊥_ ≅ 2.050, A_⊥_≅ 23 G), can also be undoubtedly assigned to isolated copper centers in slightly different environments. The last two components with very low signal intensities can be assigned to the electrons trapped at the oxygen vacancies (R‐1, g = 2.002)^[^ ^21^, ^25^ ^]^ and the Ti(III) centers present in the TiO_2_ matrix (Ti‐1, g_||_ ≅  1.924, and g_⊥_ ≅ 1.945).^[^ ^24a^ ^]^ Similarly, EPR analysis of the a‐TiO_2_‐Fe**^SA^** sample (Figure S11, Supporting Information) revealed a main signal that is typically attributed to isolated Fe(III) ions at the surface or near the surface of TiO_2_,^[^ ^5^, ^13^, ^26^ ^]^ although a minor amount of small iron oxide‐type clusters wrapped up by the anatase matrix is also possible (for details see Note S1, Supporting Information). These EPR results for a‐TiO_2_‐Cu**^SA^** and a‐TiO_2_‐Fe**^SA^** are fully in line with the X‐ray absorption data (XANES/EXAFS), which effectively rules out the presence of larger CuO_x_ or FeO_x_ clusters and indicates that the Cu(II) and Fe(III) ions are strongly bound, rather than weakly physisorbed, to the surface of anatase TiO_2_ (for details see Note S2 and Figures S12 and S13, Supporting Information, note that due the overlap of the V K_α_ lines with the Ti K_β_ lines, XAS analysis of the vanadium‐containing sample a‐TiO_2_(400)‐V**^SA^** could not be performed).

At very low surface coverages on metal oxide supports, vanadate species are mostly present as isolated VO_x_ species in typically distorted structures between tetrahedral (VO_4_) or octahedral (VO_6_) arrangements.^[^ ^20^, ^27^ ^]^ During the EPR analysis, the vanadate species became active after the photoreduction of V(V) to V(IV) (*d* ^1^ electronic configuration). The complex EPR spectrum of the a‐TiO_2_(400)‐V**^SA^** sample recorded after 120 min of UV irradiation (Figure 2b) consists of four components, as determined by computer simulation, suggesting the presence of at least four different vanadate species in distinct local environments. The EPR signals showed nearly axial symmetry with a well‐resolved hyperfine structure owing to the interaction between the electron and nuclear (^51^ V, 99.75%, *I* = 7/2) spin magnetic moments. On the basis of the similarity of the magnetic parameters, the components can be classified into two groups (VO‐1A/B and VO‐2A/B; see also **Table** **1**): VO‐1A (≈15%) with magnetic parameters g_||_  = 1.919, A_||_ = 200.9 G and g_⊥_ = 1.981, A_⊥_ = 75.6 G, calculated g_iso_ = 1.960 and A_iso_ = 117 G; VO‐1B (≈35%) with magnetic parameters g_||_  = 1.907, A_||_ ≅ 201.2 G and g_⊥_ ≅ 1.979, A_⊥_ = 71.9 G, calculated g_iso_ = 1.955 and A_iso_ = 115 G; VO‐2A (≈25%) with magnetic parameters g_||_  = 1.950, A_||_ ≅ 170.5 G and g_⊥_ ≅ 1.971, A_⊥_ = 51.0 G, calculated g_iso_ = 1.964 and A_iso_ = 90.8 G; VO‐2B (≈25%) with magnetic parameters g_||_  = 1.938, A_| |_≅ 178.3 G and g_⊥_ ≅ 1.972, A_⊥_ = 57.3 G, calculated g_iso_ = 1.961 and A_iso_ = 97.6 G. Both centers, VO‐1 and VO‐2, represent well‐known monomeric surface vanadyl VO^2+^ species in a distorted octahedral (VO‐1) or tetrahedral (VO‐2) environment, as indicated by the values of g_iso_ and A_iso_, as well as the sharp lines with well‐resolved HFS.^[^ ^27^, ^28^ ^]^ The parameters listed in Table 1 suggest that the symmetry of the VO‐1 centers is more distorted.^[^ ^27b,c^ ^]^

**Table 1:** Parameters obtained by simulation of EPR spectra of a‐TiO_2_(400)‐V^SA^.

Species^a^ ^),b)^	g_||_	A_||_ [G]	g_⊥_	A_⊥_ [G]	I_rel_ [%]	α	($\beta_{2}^{*}$)^2^
VO‐1A	1.919	200.9	1.981	75.6	15	3.9	0.96
VO‐1B	1.907	201.2	1.979	71.9	35	4.1	0.98
VO‐2A	1.950	170.5	1.971	71.0	25	1.7	0.95
VO‐2B	1.938	178.3	1.972	57.3	25	2.1	0.95

^a)^ g, A, and the relative intensities (*I_rel_*), as well as two additional parameters $\alpha =\frac{\Delta g_{\left|\right|}}{\Delta g_{⊥}}=\frac{g_{e}-g_{\left|\right|}}{g_{e}-g_{⊥}}$, where g_*e*_ = 2.0023 (*g* for free electrons) and ($\beta_{2}^{*}$)^2^. The parameter α is related to the axial distortion of the vanadyl center, whereas the parameter ($\beta_{2}^{*}$)^2^ describes the extent of electron delocalization and was calculated from the equation ($\beta_{2}^{*}$)^2^ = $\frac{5}{12}\Delta g_{⊥}-\frac{7}{6}\Delta g_{\left|\right|}+\frac{7}{6}\frac{A_{⊥}-A_{\left|\right|}}{P}$,^[^ ^29^ ^]^ where *P*, a parameter describing the relationship between the Bohr and nuclear magnetons, was set as 140 G at 128∙10^─4^ cm^─1^ (calculated for averaged *g_iso_* = 1.96).^[^ ^30^ ^]^ A lower ($\beta_{2}^{*}$)^2^ value indicates delocalization of the electron onto the oxygen ligand and a higher degree of covalency of the V─O bond.

### 2.2 Photocatalytic Performance

**Figure 3:**
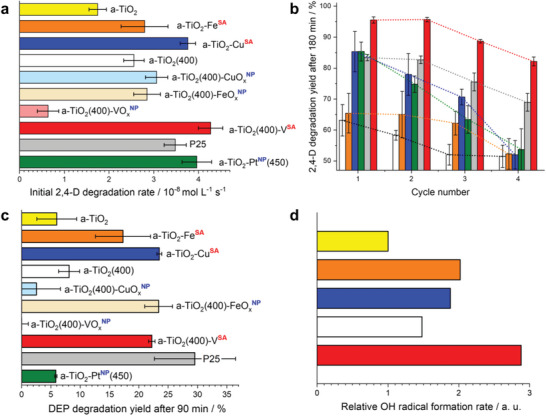
Photocatalytic performance of TiO_2_ photocatalysts modified with single‐atom (^SA^) catalytic sites under simulated sunlight. The photocatalytic activity is shown with respect to that of various benchmark photocatalysts: pristine anatase TiO_2_ (a‐TiO_2_), anatase powder pre‐heated at 400 °C (a‐TiO_2_(400)), commercial P25 (anatase/rutile) photocatalyst, and anatase photocatalysts modified with platinum or metal‐oxide (CuO_x_, FeO_x_, or VO_x_) nanoparticles (^NP^); results are shown for the optimized loadings affording the highest activity; the data for materials modified with CuO_x_ and FeO_x_ nanoparticles are taken from our previous work.^[^ ^19^ ^]^ a) Initial (after 60 min) photocatalytic degradation rate of 2,4‐dichlorophenoxyacetic acid (2,4‐D). b) Degradation yield of 2,4‐D after prolonged (180 min) irradiation during four consecutive degradation cycles. c) Degradation yield of diethyl phthalate (DEP) after 90 min irradiation. d) Relative formation rate of OH radicals, estimated by oxidation of terephthalic acid to hydroxyterephthalic acid over 30 min. Error bars were constructed using 2σ values (σ  = standard deviation; confidence interval of ≈95%).

The photocatalytic performance of the optimized a‐TiO_2_‐Cu**^SA^**, a‐TiO_2_‐Fe**^SA^**, and a‐TiO_2_(400)‐V**^SA^** photocatalysts was investigated in comparison to that of various benchmark photocatalysts: pristine anatase TiO_2_ (a‐TiO_2_), anatase powder pre‐heated at 400 °C (a‐TiO_2_(400)), commercial P25 (anatase/rutile) photocatalyst, and anatase photocatalysts modified with platinum or metal oxide (CuO_x_, FeO_x_, or VO_x_) nanoparticles (NP) obtained in our previous work.^[^ ^19^ ^]^ Note that the SAC‐modified photocatalysts and all benchmark photocatalysts were optimized to achieve the highest activity in terms of the co‐catalyst loading and other process parameters (such as the amount of photocatalyst used during the experiment; for details see the, Section S1.5 and Figures S1 and S2, Supporting Information). The data obtained for the respective best‐performing materials under the optimized conditions are shown in **Figure** **3**.

First, the photocatalytic activity in photodegradation of 2,4‐dichlorophenoxyacetic acid (2,4‐D) as a test pollutant was investigated under simulated solar irradiation. The initial degradation rates (Figure 3a) at the SAC‐modified a‐TiO_2_‐Cu**^SA^**, a‐TiO_2_‐Fe**^SA^**, and a‐TiO_2_(400)‐V**^SA^** photocatalysts were higher than those of the unmodified counterparts by factors of ≈2.1, ≈1.6, and ≈1.7, respectively. The photocatalytic degradation of the 2,4‐D test pollutant, followed by changes in the UV–vis electronic absorption, led to its complete mineralization, as evident from the corresponding analyses of the total organic carbon content (TOC, Figure S14, Supporting Information). Notably, except for a‐TiO_2_‐Fe**^SA^**, the SAC‐modified photocatalysts exhibited significantly higher initial degradation rates than the TiO_2_ materials modified with the corresponding metal oxide nanoparticles. It is assumed that the advantage of SACs compared to the corresponding metal‐oxide nanoparticles is derived mainly from the negligible parasitic light absorption of the former. The electronic absorption spectra of the SAC‐modified samples obtained using diffuse reflectance spectroscopy showed negligible differences compared to those of the unmodified materials (Figure S15, Supporting Information). The most drastic difference in activity was observed for a‐TiO_2_(400)‐V**^SA^**, for which the activity was ≈6.5 times higher than that of the material modified with VO_x_ nanoparticles (a‐TiO_2_(400)‐VO_x_ **^NP^**), which is ascribed to the blocking effect of the VO_x_ nanoparticles on the absorption of UV light by TiO_2_. The significant optical absorption of the VO_x_ nanoparticles is apparent from the absorption shoulder extending to >500 nm (see Figure S15b, Supporting Information), in contrast with the absorption range of isolated vanadate SACs, which are known to absorb only below 320 nm.^[^ ^31^ ^]^

The vanadate‐modified SAC photocatalyst (a‐TiO_2_(400)‐V**^SA^**) exhibited the highest degradation rates of all the studied materials, outperforming even platinized TiO_2_ and commercial P25 powder, which generally exhibit superior performance in the photocatalysis of various reactions.^[^ ^32^ ^]^ The outstanding performance of a‐TiO_2_(400)‐V**^SA^** is highlighted further when considering the overall degradation yield after 180 min of irradiation and the operational stability after several photodegradation cycles with the same initial pollutant concentration (Figure 3b). In this context, it should be noted that while anatase powder modified with vanadate (a‐TiO_2_(400)‐V**^SA^**) consistently outperformed P25 (anatase/rutile mixture) in terms of both the initial degradation rate and degradation yield (Figure 3a,b), the modification of P25 with vanadate using identical protocols did not result in improved performance, whereas in the case of modified rutile TiO_2_, even an inhibitory effect was observed (Figure S16, Supporting Information). Thus, the beneficial effects of vanadate modification are highly dependent on the starting TiO_2_ phase; the anatase phase is preferable. In other words, the present results suggest that when attempting to prepare a photocatalyst that outperforms P25 by using SACs, it is better not to use P25 as the starting material.

Notably, the photoactivity of all the studied materials declined over four consecutive photodegradation cycles (Figure 3b). However, a similar decline was also observed with pristine and platinized TiO_2_ or P25, suggesting that apart from the expected partial loss of the co‐catalyst, for example, via reductive dissolution in the case of Cu(II) and Fe(III), other factors also play a role in the performance deterioration. These factors might include the accumulation of reaction intermediates that effectively inhibit the degradation reaction at the photocatalyst surface. Indeed, the relative decrease in activity during cycling of the best‐performing a‐TiO_2_(400)‐V**^SA^** photocatalyst and P25 is very similar, which indicates that the loss of the co‐catalyst is not a major problem. For practical applications, operational stability issues should be addressed by engineering efforts to achieve process intensification using optimized operational parameters and photoreactor designs.^[^ ^33^ ^]^

Because the specific performance of TiO_2_‐based photocatalysts is known to be highly substrate dependent, two other photocatalytic reactions were investigated to obtain a more accurate assessment of the advantages and limitations of the developed SAC‐modified photocatalysts. First, the photodegradation of diethyl phthalate (DEP), a toxic pollutant that does not adsorb well onto TiO_2_ was investigated; the kinetics of DEP degradation are typically significantly slower than those of 2,4‐D.^[^ ^34^ ^]^ Again, the photocatalytic degradation rate was enhanced for the SAC‐modified materials a‐TiO_2_‐Cu**^SA^**, a‐TiO_2_‐Fe**^SA^**, and a‐TiO_2_(400)‐V**^SA^** by factors of ≈3.9, ≈2.9, and ≈2.8, respectively, compared with that of the corresponding unmodified TiO_2_ samples (Figure 3c). Moreover, with the exception of a‐TiO_2_‐Fe**^SA^**, all SAC‐modified materials outperformed the corresponding nanoparticle‐modified samples. The difference was most striking in the case of the material modified with VO_x_ nanoparticles (a‐TiO_2_(400)‐VO_x_ **^NP^**), which showed negligible DEP photodegradation rates, in contrast with the very high activity of the SAC‐modified material a‐TiO_2_(400)‐V**^SA^**. Second, the rate of formation of the OH radical, determined by the oxidation of terephthalic acid to hydroxyterephthalic acid, was also significantly enhanced in all the SAC‐modified samples, where the highest rate was obtained with a‐TiO_2_(400)‐V**^SA^** (Figure 3d). These results show that the beneficial effect of SAC modification on the efficacy of anatase TiO_2_ for the photocatalytic degradation of pollutants is not confined to specific oxidizable test substrates, but is rather general. This finding is also in line with our assumption that the presence of reducible SACs affects the overall photocatalytic reactivity predominantly by inducing changes in the reductive pathway induced by the photogenerated electrons, i.e., by trapping photogenerated electrons at the SAC‐induced surface reactive sites, enhancing the reaction with dioxygen, and/or catalyzing the subsequent redox transformations of reactive oxygen species (e.g., superoxide anion radicals and H_2_O_2_)^[^ ^35^ ^]^ formed during the photocatalytic process.

### 2.3 In Situ EPR Investigations

**Figure 4:**
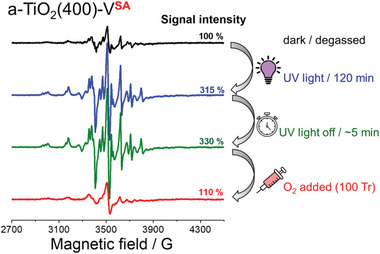
In situ X‐band EPR spectra of a‐TiO_2_(400) ‐V^SA^. EPR signal of vanadate species upon UV light‐induced reduction and subsequent addition of oxygen. For the time‐resolved signal development during the initial photoreduction, see Figure S17 (Supporting Information).

To obtain deeper insight into the mechanism of photoactivity enhancement in SAC‐modified photocatalysts, a series of in situ EPR experiments was carried out to directly observe the changes in the oxidation state of the SACs upon photoreduction and subsequent exposure to dioxygen. a‐TiO_2_(400)‐V**^SA^** and a‐TiO_2_‐Cu**^SA^** were comparatively analyzed because these materials exhibited the highest photocatalytic enhancement. Moreover, their EPR spectra could be interpreted in a straightforward manner, unlike that of a‐TiO_2_‐Fe**^SA^** (see Figure S11 and Note S1, Supporting Information). The best‐performing sample a‐TiO_2_(400)‐V**^SA^** was first evaluated. After degassing, the EPR spectrum of a‐TiO_2_(400)‐V**^SA^** showed only a weak signal from the monomeric surficial vanadyl VO^2+^ species in a distorted octahedral environment (**Figure** **4**). As expected, UV irradiation resulted in a strong increase in the signal intensity upon the reduction of EPR‐silent V(V) to EPR‐active V(IV). This indicates that the surface vanadate species in a‐TiO_2_(400)‐V**^SA^** can be effectively reduced by the photogenerated electrons in TiO_2_. Notably, the EPR signal remained virtually unchanged after irradiation was discontinued as long as the system was kept under oxygen‐free conditions. However, exposure of the a‐TiO_2_‐V^SA^ sample to 100 Torr of O_2_ at room temperature led to a strongly diminished signal with roughly the same intensity and shape as that before irradiation (Figure 4). This phenomenon demonstrates that the photoreduced surface‐bound V(IV) species are readily and quickly re‐oxidized by dioxygen. The observation that the surface vanadate species can be easily reduced by photogenerated electrons and subsequently readily re‐oxidized by oxygen suggests that under photocatalytic conditions, the vanadate species can act as a very effective single‐atom redox catalyst for one‐electron dioxygen reduction by photogenerated electrons. In turn, the faster rate of oxygen reduction by the photogenerated electrons catalyzed by vanadate SACs reduces the probability of charge recombination, leading to enhanced rates of photocatalysis.

**Figure 5:**
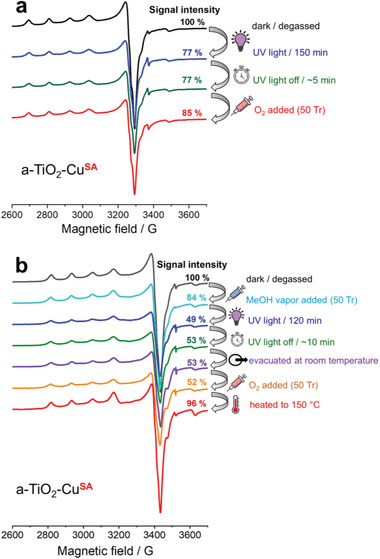
In situ X‐band EPR spectra of a‐TiO_2_‐Cu^SA^. a) EPR signal of single Cu(II) sites upon UV light‐induced reduction in the absence of methanol vapor and subsequent addition of oxygen. b) EPR signal of single Cu(II) sites upon photoreduction in the presence of methanol vapor and subsequent steps. For the time‐resolved signal development during the initial photoreduction steps, see Figures S18 and S19 (Supporting Information).

An analogous in situ EPR study was performed using the Cu(II) ion‐modified sample, a‐TiO_2_‐Cu**^SA^** (**Figure** **5**a). Upon UV irradiation, the intensity of the signal originating from the isolated Cu(II) sites decreased to ≈77% of the initial intensity, indicating the reduction of a portion of single Cu(II) ions to Cu(I) by the photogenerated electrons in TiO_2_. The EPR spectrum after UV irradiation could be simulated using the same components as described above (see Figure 2a); however, the contributions of the R‐1 and Ti‐1 centers were higher, corresponding to the electrons trapped at the oxygen vacancies^[^ ^21^, ^25^ ^]^ and Ti^3+^ centers, respectively.^[^ ^24a^ ^]^ Similar to the case of a‐TiO_2_(400)‐V**^SA^**, no change in the EPR signal was observed when irradiation was discontinued. However, in stark contrast to the behavior of a‐TiO_2_(400)‐V**^SA^,** after exposing the sample to dioxygen, the signal intensity was only partially recovered and reached only ≈85% of the initial intensity. This suggests that in contrast to the very fast re‐oxidation of V(IV) to V(V) in a‐TiO_2_(400)‐V**^SA^,** the majority of the photoreduced single Cu(I) sites in a‐TiO_2_‐Cu**^SA^** cannot be readily re‐oxidized by dioxygen. To further investigate the distinct behavior of a‐TiO_2_‐Cu**^SA^** in more detail, a series of in situ EPR experiments was conducted, in which the initial photoreduction was carried out in the presence of methanol vapor (50 Torr) (Figure 5b). Because methanol can act as an efficient scavenger for the photogenerated holes in TiO_2_, it was assumed that the yield of reduced Cu(I) sites might be higher. Indeed, in the presence of methanol vapor, the Cu(II) signal was reduced to nearly half of the initial intensity. After evacuating the EPR tube and adding O_2_, the signal remained practically the same (≈52%), which again confirms that the reduced Cu(I) species in a‐TiO_2_‐Cu**^SA^** did not react readily with dioxygen at room temperature. However, after heating the sample to 150°C in the presence of O_2_, the initial signal of Cu(II) was almost completely (≈96%) recovered.

Several points are worth noting in this context. First, the absence or presence of an additional hole scavenger (methanol) strongly affected the yield of the photoreduced Cu(I) species. In this context, we note that almost complete reduction of Cu(II) to Cu(I) was observed during EPR analysis of the a‐TiO_2_‐Cu**^SA^** photocatalyst in the liquid water/methanol mixture (Figure S20, Supporting Information). The strong EPR signal (g_||_  ≅  2.348, A_||_ ≅ 120 G, and g_⊥_ ≅ 2.065, A_⊥_ – not resolved) visible prior to UV irradiation completely disappeared after only 15 min of UV irradiation. Simultaneously, the intensities of the EPR signals characterized by *g* = 2.004 (i.e., electrons trapped at oxygen vacancies, R‐1),^[^ ^21^, ^25^ ^]^ and g_||_ ≅ 1.935 and g_⊥_ ≅ 1.970 (i.e., Ti^3+^ centers, Ti‐1)^[^ ^24a^ ^]^ increased significantly. Moreover, the Cu(II) signal disappeared completely, although oxygen was not removed from the mixture during this experiment, which again confirms that the re‐oxidation of reduced Cu(I) by oxygen is very slow compared to the reduction. Second, the observation of the nearly complete recovery (≈96%) of the Cu(II) signal after increasing the temperature to 150 °C indicates not only that the Cu(II)/Cu(I) single‐atom sites are stable during the reduction/re‐oxidation cycle, but also that the re‐oxidation of Cu(I) to Cu(II) in a‐TiO_2_‐Cu**^SA^** by dioxygen requires a mild thermal activation step. This, third, is in stark contrast not only to the very fast room‐temperature re‐oxidation of V(IV) to V(V) by O_2_ in a‐TiO_2_(400)‐V**^SA^**, but also to the behavior of recently reported anatase TiO_2_ in which single Cu(II/I) ions occupy the Ti vacancy sites, which induced fast re‐oxidation of reduced Cu(I) sites by oxygen.^[^ ^6d^ ^]^ This indicates that the developed a‐TiO_2_‐Cu**^SA^** with surface‐grafted Cu(II) SACs is distinct from the material reported by Lee et al. in which the Cu(II) ions act as substitutional dopants, suggesting that the reactivity of Cu(II) SACs toward oxygen is strongly dependent on the local bonding environment of Cu(II) in anatase TiO_2_. Fourth, these results bring into question the origin of the significant enhancement of the photocatalytic degradation rates at a‐TiO_2_‐Cu**^SA^** (see Figure 3). Because the kinetics of the re‐oxidation of Cu(I) to Cu(II) in a‐TiO_2_‐Cu**^SA^** is rather sluggish, the catalysis of dioxygen reduction (the primary reaction in the reductive pathway), as in the case of a‐TiO_2_(400)‐V**^SA^**, is unlikely to be the reason. The present EPR analysis demonstrates that the Cu(II) SAC sites can effectively trap photogenerated electrons, thereby improving the charge separation yield. However, an efficient photocatalytic process requires continuous re‐oxidization of the Cu(I) sites generated after reduction by photogenerated electrons during the photocatalytic operation on a timescale kinetically similar to electron trapping at the Cu(II)/Cu(I) sites. In this context, it is important to realize that O_2_ is not the only oxidizing agent available for the re‐oxidation of Cu(I) in conventional photocatalytic degradation processes. In particular, it is well established that hydrogen peroxide is formed during the photocatalysis of TiO_2_, especially when organic compounds act as hole scavengers, as is the case in the pollutant degradation reactions studied herein.^[^ ^35^, ^36^ ^]^ Under such conditions, significant amounts of H_2_O_2_ can be formed via various reactions in the reductive pathway, initiated by the reduction of dioxygen to the superoxide anion radical $O_{2}^{•-}$ by the photogenerated electrons ($e_{\mathrm{CB}}^{-}$) in the conduction band of TiO_2_:

(1) $$O_{2}+e_{\mathrm{CB}}^{-}\to O_{2}^{•-}$$

(2) $$O_{2}^{•-}+H^{+}\to {\mathrm{HO}}_{2}^{•}$$

(3) $${\mathrm{HO}}_{2}^{•}+{\mathrm{HO}}_{2}^{•}\to H_{2}O_{2}+O_{2}$$

(4) $$O_{2}^{•-}+{\mathrm{HO}}_{2}^{•}\to O_{2}{+\mathrm{HO}}_{2}^{-}$$

(5) $${\mathrm{HO}}_{2}^{-}+H^{+}\to H_{2}O_{2}$$

(6) $$O_{2}^{•-}+2H^{+}+e_{\mathrm{CB}}^{-}\to H_{2}O_{2}$$

In particular, the formation of H_2_O_2_ upon disproportionation of the protonated superoxide anion radical (3) is known to occur readily (*k*_disp_ = 5 × 10^6^ m ^−1^ s^−1^ at pH 6).^[^ ^37^ ^]^ Consequently, the H_2_O_2_ formed during photocatalysis can undergo the Fenton reaction, which is well‐known to proceed highly efficiently (*k* = 1.0 × 10^4^ m ^−1^ s^−1^)^[^ ^38^ ^]^ with Cu(I) ions:

(7) $$\mathrm{Cu}\left(I\right)+H_{2}O_{2}\to \mathrm{Cu}\left(\mathrm{II}\right)+{}^{•}\mathrm{OH}+HO^{-}$$

It is, therefore, highly likely that the Cu(I) formed during the photocatalytic cycle at a‐TiO_2_‐Cu**^SA^** can be re‐oxidized to Cu(II) by H_2_O_2_, which explains the increased photodegradation activity of a‐TiO_2_‐Cu**^SA^** compared to that of the Cu‐free benchmark TiO_2_ material (Figure 3). Moreover, Cu(II) can be reduced upon reaction with H_2_O_2_, albeit with a relatively much lower rate constant (*k* = 4.6 × 10^2^ m ^−1^ s^−1^).^[^ ^38^ ^]^ Hence, in the presence of the H_2_O_2_ intermediate, Cu(I) can also be formed via a non‐photochemical pathway:

(8) $$\mathrm{Cu}\left(\mathrm{II}\right)+H_{2}O_{2}\to \mathrm{Cu}\left(I\right)+{\mathrm{HO}}_{2}^{•}+HO^{-}$$

However, because reaction (8) is much slower than reaction (7), it can be assumed that without irradiation, the predominant oxidation state of Cu is +2, and most of the Cu(I) species are formed via reduction by the photogenerated electrons. In other words, the enhanced activity of a‐TiO_2_‐Cu**^SA^** can be attributed to a distinct type of photo‐Fenton reaction,^[^ ^38^, ^39^ ^]^ which proceeds on the surface of anatase TiO_2_ modified with Cu(II) SACs through a combination of effective trapping of the photogenerated electrons at Cu(II), followed by the Fenton step (Equation 7), i.e., the reaction of photoreduced Cu(I) with H_2_O_2_ to regenerate Cu(II). The spin‐trapping EPR experiments confirmed the formation of hydroxyl radicals in the UV‐irradiated aqueous suspensions of all studied photocatalysts and of both ^•^OH and carbon‐centered radicals in the case of the water/methanol mixtures (for details see Note S3, Figure S21 and Table S2, Supporting Information). Direct detection of superoxide anion radicals was not successful, most likely because DMPO–OOH adducts are known to be very unstable and spontaneously decompose into non‐radical species and DMPO–OH.^[^ ^40^ ^]^

### 2.4 Theoretical Calculations

**Figure 6:**
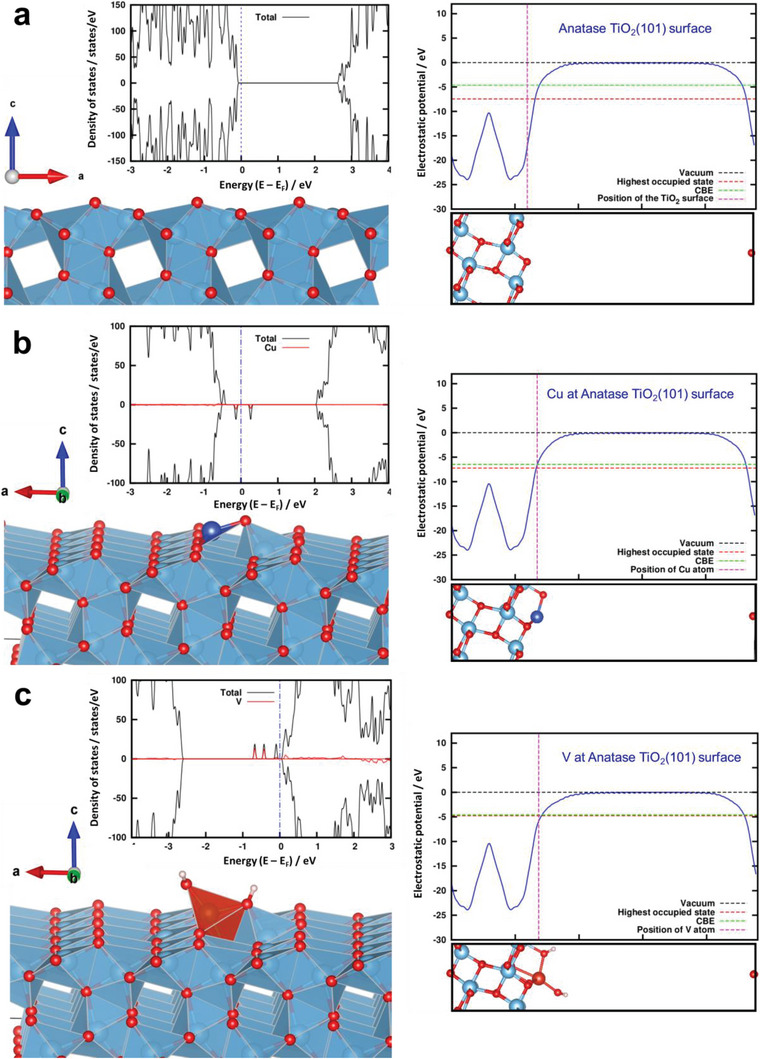
Periodic DFT calculations. Models of atomic structure, calculated density of states plots, and local electrostatic potential profiles of anatase TiO_2_(101) surfaces without a) and with surface‐grafted Cu(II) b) and vanadate species c).

To elucidate the cause of the striking difference in the reactivity of the electrons trapped in the SAC‐induced states in a‐TiO_2_(400)‐V**^SA^** versus a‐TiO_2_‐Cu**^SA^** with dioxygen, a set of DFT calculations was performed (for details see Supporting Information, 3. Computational details). The (101) surface of anatase TiO_2_ was used in the calculations because it is the most stable and prevalent facet in the small TiO_2_ crystallites present in the developed materials.^[^ ^41^ ^]^ The results are summarized in **Figure** **6**. Grafting of Cu(II) and vanadate species onto the anatase (101) surface induced the formation of highly localized intragap states in both cases. The intragap states are found in the same spin state in each case, which indicates that no spin exchange energy is involved in the bandgap. The calculated plots of the density of states indicate that these intragap states lie deeper below the conduction band edge of anatase TiO_2_ in the case of Cu than in the case of vanadate. To obtain a more quantitative picture of the energetic positions of the intragap states, the local electrostatic potential at the surface of the materials was modelled. In general, the position of the conduction band edge (CBE) of anatase TiO_2_ in all materials is determined by the net surface charge, which is mainly governed by the adsorption of protons and hydroxyl ions at the TiO_2_ surface. Therefore, the positions of the band edges strongly depend on the pH of the aqueous solution. Herein, it is assumed that because of the relatively low surface concentration of Cu(II) or vanadate ions, the CBE positions of all of the studied materials coincide with the typical position of the CBE of pristine anatase TiO_2_, which is ca. –0.6 V versus NHE at pH 7.^[^ ^42^ ^]^ The relative position of the intragap states with respect to the CBE was then estimated from the plots of the local electrostatic potential by taking the difference between the highest occupied state and the CBE. Based on this analysis, it was estimated that the vanadate‐induced electronic states were positioned immediately below (0.13 eV) the CBE of anatase TiO_2_. In contrast, the Cu(II)‐induced intragap states lie much deeper, centered at ≈0.73 eV below the CBE of anatase TiO_2_.

**Figure 7:**
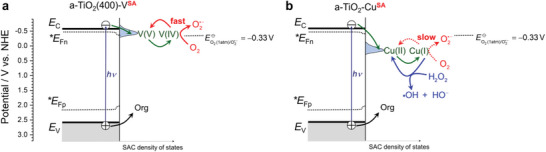
Different mechanisms of enhancement of photocatalytic degradation rate on anatase TiO_2_ surface modified with different SACs. a) At a‐TiO_2_(400)‐V^SA^, the vanadate‐induced interfacial states are just below (by 0.13 eV) the conduction band edge of anatase TiO_2_ (–0.6 V vs NHE at pH 7). After trapping the photogenerated electrons at surface V(V) sites, the reaction of reduced V(IV) species with O_2_ is very fast, which directly translates to diminished electron‐hole recombination and enhanced degradation rates. b) At a‐TiO_2_‐Cu^SA^, the presence of Cu(II) induces relatively deeper (by 0.73 eV) electronic states with respect to the conduction band edge of anatase TiO_2_. The photogenerated electrons trapped at Cu(I) states do not react readily with O_2_, but instead undergo very effective Fenton reaction (Equation 7) with the H_2_O_2_ produced during photocatalysis. $E_{O_{2}\left(1\;\mathrm{atm}\right)/O_{2}^{•-}}^{=}-0.33\;V$is the standard reduction potential for one‐electron reduction of O_2_ to the superoxide anion radical (assuming the standard state with unit fugacity, i.e., the partial pressure of O_2_ ∼ 1 atm).^[^ ^43^ ^]^ *E*_C_, *E*_V_, *E_Fn_, and *E_Fp_ stand for the conduction and valence band edge and the quasi‐Fermi levels of electrons and holes, respectively. For details see the text.

Based on these differences, the mechanistic differences in the photocatalytic enhancements of a‐TiO_2_(400)‐V**^SA^** and a‐TiO_2_‐Cu**^SA^** can be rationalized (**Figure** **7**). In a‐TiO_2_(400)‐V**^SA^**, the photogenerated electrons trapped at V(IV) sites just below the CBE of anatase TiO_2_ at ≈–0.5 V vs NHE (at pH 7) have a reduction potential negative enough to react very efficiently with O_2_ to form the superoxide anion radical (standard reduction potential $E_{O_{2}\left(1\;\mathrm{atm}\right)/O_{2}^{•-}}^{=}-0.33\;V$ vs NHE). This explains the very fast recovery of V(V) upon exposure of the reduced V(IV) sites to O_2_ (Figure 4). In other words, the vanadate species act as an effective SAC for the reduction of O_2_ to $O_{2}^{•-}$ by photogenerated electrons. The faster consumption of photogenerated electrons translates into diminished recombination of electron‐hole pairs and is the basis for the effective enhancement of the photocatalysis at a‐TiO_2_(400)‐V**^SA^** (Figure 7a). This situation is very different for a‐TiO_2_‐Cu^SA^ (Figure 7b) as the Cu‐induced intragap states are centered at ca. +0.1 V vs NHE (pH 7). Compared to a‐TiO_2_(400)‐V**^SA^**, the thermodynamic driving force for the direct reduction of O_2_ is therefore diminished by ≈0.6 V. This leads to very low reactivity of the electrons trapped at the surface Cu(I) sites toward dioxygen unless the temperature is increased (see Figure 5). As discussed above, the enhancement of photocatalysis at a‐TiO_2_‐Cu**^SA^** can be ascribed to the combined effect of the fast trapping of photogenerated electrons at Cu(II), followed by the Fenton reaction, i.e., the reaction of reduced Cu(I) with H_2_O_2_ to regenerate Cu(II) (Equation 7).

### 2.5 Mechanisms of Activity Enhancement

In summary, while both Cu(II) and vanadate act as effective SACs for enhancing the photocatalytic degradation rate, the mechanisms of enhancement are similar and different in each case. The similarity mainly lies in the fact that both Cu(II) and vanadate SACs are easily reducible, and thus can effectively trap electrons photogenerated in TiO_2_ and enhance the initial charge separation. The positive effect of all SACs on primary charge separation is supported by our observation of partial quenching of the photoluminescence band centered in the green region (≈2.35 eV, ≈528 nm) of the optical spectrum (see Figure S22, Supporting Information), which has been ascribed to the radiative recombination of conduction band electrons with trapped holes.^[^ ^44^ ^]^ The key difference is the reactivity of the reduced catalyst toward dioxygen. Vanadate is easily re‐oxidized by O_2_ and acts as a SAC for the direct reduction of O_2_ to superoxide anion radicals, i.e., for one of the primary reaction steps in the photocatalytic degradation mechanism. In contrast, the reduced Cu(I) does not react with dioxygen, but with H_2_O_2_, a typical intermediate of the photocatalytic degradation mechanism. Hence, the Cu(II) ions in a‐TiO_2_‐Cu**^SA^** act effectively as SACs for a secondary reaction in the photodegradation mechanism, namely, a distinct type of photo‐Fenton process, converting H_2_O_2_ to hydroxyl radicals and hydroxyl ions. Notably, the mechanistic differences related to the catalytic enhancement of the rate of dioxygen reduction at different SACs identified by the present EPR investigations were further supported by analysis of the photogenerated electron lifetimes derived from the photopotential measurements recorded in the absence and presence of dioxygen (see Figures S23 and S24, Supporting Information). The results of the electron lifetime measurements strongly corroborate our conclusion about the fundamental difference in the catalytic enhancement of dioxygen reduction in photocatalysts modified with vanadate SACs as compared to those with Cu(II) SACs, and indicate that photocatalysts modified with Fe(III) SACs function similarly to the congeners with Cu(II), i.e. , the rate of primary O_2_ reduction is not significantly enhanced (for details see Note S4, Supporting Information). This suggests that the mechanism of photoactivity enhancement in Fe(III) SAC‐modified TiO_2_ might be similar to that in a‐TiO_2_‐Cu**^SA^**, which is expected because both Cu(II/I) and Fe(III/II) are prone to Fenton‐like chemistry.^[^ ^38^ ^]^

In this context, we recall again the superior performance of a‐TiO_2_(400)‐V**^SA^** with respect to the overall photocatalytic degradation yield and operational stability over several photodegradation cycles (Figure 3b). The present results suggest that this superior performance is directly related to the ability of the vanadate SACs to catalyze the primary reaction with dioxygen. In contrast, a‐TiO_2_‐Cu**^SA^** exhibits both lower activity and stability. This can be ascribed to the poor reactivity of the reduced Cu(I) SAC sites with dioxygen, which results in a significant portion of the SAC being in a reduced Cu(I) state during the photocatalytic cycle. This, in turn, not only limits the enhancement of charge separation because the trapping of photogenerated electrons is less effective, but also increases the tendency of copper ions to leach into the solution because the propensity of first‐row transition metal ions to dissolve in aqueous solutions is known to increase significantly upon reduction. Therefore, it is concluded that the ability of SACs to provide fast catalytic turnover in reactions with dioxygen is highly beneficial for the overall performance of SAC‐modified TiO_2_ photocatalysts for the oxidative photocatalytic degradation of organic compounds.

Based on this knowledge, we hypothesized that the superior oxygen reduction kinetics of TiO_2_ modified with vanadate SACs might be highly beneficial for photocatalytic transformations beyond the oxidative degradation of organic pollutants in aqueous solutions. To this end, the activity and selectivity of a‐TiO_2_(400)‐V**^SA^** was evaluated in the gas‐phase photocatalytic conversion of NO_2_ (for details see Section S1.5 and Figure S25, Supporting Information). Indeed, the overall activity of a‐TiO_2_(400)‐V**^SA^** in the conversion of NO_2_ was enhanced by a factor of ≈3 compared to that of pristine a‐TiO_2_(400) (**Table** **2**), which can be ascribed to the effective de‐bottlenecking of the oxygen reduction pathway. Moreover, the vanadate SACs partially change the selectivity of the process, as a portion of NO_2_ (≈33%) was reduced to NO, a process that does not occur in unmodified TiO_2_ where all of the NO_2_ is converted to nitrate. These results suggest that although nitrate is still the dominant product, some of the photogenerated electrons trapped at the reduced V(IV) sites react directly with NO_2_ to produce NO, most likely via nitrite intermediates. These results confirm that the highly beneficial role of vanadate SACs in TiO_2_ for photoactivity enhancement is not confined to the photodegradation of organic compounds, but is a rather general feature that can be utilized in any photocatalytic conversion that relies on the rapid turnover of electron acceptors that react readily with reduced vanadate SACs.

**Table 2:** Photocatalytic conversion of NO_2_.

Sample^a)^	NO_2_ conversion	Selectivity toward NO	Selectivity toward nitrate
a‐TiO_2_(400)	3.5%	0	100%
a‐TiO_2_(400)‐V**^SA^**	10.1%	33%	67%

^a)^ For raw measurement data, see Figure S25 (Supporting Information).

## 3 Conclusion

Novel, highly active photocatalysts based on anatase TiO_2_ modified with various single‐atom catalysts were developed for the light‐driven aerobic degradation of pollutants. The study provides valuable insights into the mechanistic diversity of different SACs and important design principles for the future development of SAC‐based photocatalysts with enhanced performance. Specifically, our findings demonstrate that SACs based on low‐cost, non‐noble transition metals (such as Cu, Fe, and vanadate ions) enable remarkable enhancement of the rate of photocatalysis. The most active anatase TiO_2_ modified with vanadate SACs outperforms not only TiO_2_ modified with the corresponding metal oxide nanoparticles, but also state‐of‐the‐art benchmark photocatalysts such as platinized TiO_2_ and commercial P25 powder. Notably, in situ electron paramagnetic resonance (EPR) spectroscopy and theoretical calculations demonstrate that the mechanism of activity enhancement differs significantly for the best‐performing photocatalysts modified with Cu and vanadate SACs, particularly with respect to the rate of catalysis of dioxygen reduction by electrons that are photogenerated in TiO_2_ and extracted to the SAC. Based on the superior performance of TiO_2_ modified with vanadate SACs enabled by the fast kinetics of dioxygen reduction, it is concluded that introducing SAC‐induced intragap surface states that are rather shallow, i.e., positioned just below the conduction band edge of anatase TiO_2_, is crucial in designing highly effective SAC‐modified TiO_2_ photocatalysts. This design feature enables both effective extraction of photogenerated electrons and fast catalytic turnover in the reduction of dioxygen. The faster consumption of photogenerated electrons directly translates into diminished recombination, which makes such SAC‐modified photocatalysts examples par excellence of highly effective photoconversion systems with kinetically enhanced charge separation.^[^ ^17^, ^45^ ^]^ Thus, this study establishes a new class of highly efficient TiO_2_‐based photocatalysts for light‐driven aerobic oxidation and provides essential design guidelines for developing more efficient SAC‐based photocatalysts.

## Conflict of Interest

The authors declare no conflict of interest.

## Supporting information

Supporting Information

## Data Availability

The data that support the findings of this study are openly available in Zenodo at https://doi.org/10.5281/zenodo.7989199, reference number 7989199.
