# Supplementary material for: Enhancing Photocatalysis: Understanding the Mechanistic Diversity in Photocatalysts Modified with Single‐Atom Catalytic Sites
Source: Adv Sci (Weinh). 2023 Oct 27;10(35):2303571. doi: 10.1002/advs.202303571 (PMC10724417; doi:10.1002/advs.202303571)
Supplement: Supplementary file 1 — Supporting Information [file ADVS-10-2303571-s001.pdf]

ADVANCED SCIENCE
Open Access

Supporting Information

for *Adv. Sci.*, DOI 10.1002/advs.202303571

Enhancing Photocatalysis: Understanding the Mechanistic Diversity in Photocatalysts Modified with Single-Atom Catalytic Sites

Krzysztof Kruczała, Susann Neubert, Kapil Dhaka, Dariusz Mitoraj, Petra Jánošíková, Christiane Adler, Igor Krivtsov, Julia Patzsch, Jonathan Bloh, Johannes Biskupek, Ute Kaiser, Rosalie K. Hocking, Maytal Caspary Toroker* and Radim Beranek**

Supporting Information

Enhancing Photocatalysis: Understanding the Mechanistic Diversity in Photocatalysts Modified with Single-Atom Catalytic Sites

1. Experimental Section

1.1 Materials

Anatase TiO_2 powder (Hombikat UV 100, anatase, Sachtleben, Germany, specific surface area Brunauer-Emmett-Teller (BET) ~ 300 m^2/g, crystallite size < 10 nm), P25 TiO_2 (anatase/rutile) (Evonik, Germany), rutile TiO_2 ("Nano-Rutil" E3-692-011-009, Sachtleben, Germany), $Fe(NO_3)_3{\cdot}9H_2O$ (Riedel-de-Haën), $Cu(NO_3)_2{\cdot}3H_2O$ (Sigma-Aldrich), NH_4VO_3 (Sigma-Aldrich), H_2PtCl_6 (Sigma-Aldrich), methanol (VWR BDH Prolabo), 2,4-dichlorophenoxyacetic acid (2,4-D, Sigma-Aldrich), terephthalic acid (Sigma-Aldrich), and diethyl phthalate (Sigma-Aldrich) were used as received. For all synthetic reactions and measurements demineralized water cleaned by Siemens Ultra Pure Water Systems (conductivity: 0.055 µS/cm, TOC content: < 1 ppb, endotoxins: < 0.001 EU/m) was used.

1.2 Synthesis and optimization of a-TiO_2-Cu and a-TiO_2-Fe samples

Initially, a series of a-TiO_2-Cu and a-TiO_2-Fe samples were prepared *via* impregnation and drying technique. A suspension of TiO_2 (anatase, 2.0 g) and distilled water (50 mL) was sonicated for 5 min. Afterwards, the suspension was thoroughly stirred and the appropriate amount of $Cu(NO_3)_2{\cdot}3H_2O$ or $Fe(NO_3)_3{\cdot}9H_2O$ (0.1-2.0 mmol/L) was added to the suspension. The suspension was stirred for a further 24 h at room temperature and, subsequently, centrifuged at 4000 rpm for 15 min. The product was dried at 80 °C for 3 h followed by heating at different temperatures for 1 h. The SAC-modified samples optimized for the highest photoactivity in degradation of 2,4-D (for optimization studies see **Figure S1**) used in this study were prepared under the following conditions: 0.5 mmol/L precursor concentration and 120 °C heating temperature for a-TiO_2-Cu^{SA} (Cu amount: 0.08 wt% measured by ICP-OES), and 1.0 mmol/L and 120 °C for a-TiO_2-Fe^{SA} (Fe amount: 0.11 wt% measured by ICP-OES).

1.3 Synthesis and optimization of a-TiO_2(400)-V^{SA} and samples a-TiO_2(400)-VO_x^{NP}

Hombikat UV 100 was first pre-heated for 3 h at 400 °C (a-TiO_2(400)) in a muffle oven (Carbolite ELF 11/23 box furnace). A suspension of 50 mL distilled H_2O and 2 g of a-TiO_2(400)

was sonicated for 5 min. The pH of the suspension was adjusted to pH ~ 2 using 0.1 M HNO_3 and different amount of NH_4VO_3 (0.1 mM – 10 mM) was added to the suspension and stirred overnight. The suspension was centrifuged for 10 min at 4000 rpm and dried in an oven for 3 h at 80 °C under atmospheric pressure. The best-performing dried photocatalysts were then calcined in a muffle oven at a temperature of 450 ˚C for 3 h, which slightly improved the photoactivity. The best-performing SAC-modified sample a-TiO_2(400)-V^{SA} (V amount: 0.03 wt% measured by ICP-OES) optimized for the highest photoactivity in degradation of 2,4-D (for optimization studies see **Figure S2**) was prepared under following conditions: 0.1 mmol/L precursor concentration, 450 °C heat treatment. For comparison, a sample modified with VO_x nanoparticles (a-TiO_2(400)-VO_x^{NP}) was prepared using a precursor solution with the concentration of NH_4VO_3 increased by the factor of 100 (10 mM).

1.4 Synthesis of benchmark platinized a-TiO_2-Pt^{NP}(450) samples

Platinized anatase TiO_2 samples were prepared *via* a photodeposition technique. Hombikat UV 100 (1.0 g) was suspended in $H_2PtCl_6 \cdot 6H_2O$ (18 mL, 1 mmol/L) and methanol (2 mL). The suspension was thoroughly stirred and irradiated under a 150 W Xe-lamp (LOT Oriel) equipped with a heat-absorbing filter (KG3, Schott, ~1 sun) for different times (5, 10, 15, 20, 30, and 60 min). Afterwards, the suspension was centrifuged at 4000 rpm for 10 min. The product was dried for 3 h at 80 °C and calcined for 1 h at 450 °C in a muffle oven (Carbolite ELF 11/23 box furnace). The best-performing platinized sample a-TiO_2-Pt^{NP}(450) was obtained after 20 min photodeposition (Pt amount: 0.32 wt% measured by ICP-OES).

1.5 Photocatalytic activity tests

For photocatalytic degradation of 2,4-dichlorophenoxyacetic acid (2,4-D) and diethyl phthalate (DP), an aqueous solution of the pollutant (2.5×10^{-4} mol/L, 25 mL) was added into a borosilicate glass cell with the amount of photocatalyst optimized for the highest reaction rate (25 - 50 mg, *i.e.*, 1-2 g/L for different photocatalyts). The obtained suspension was sonicated for 5 min. Subsequently, the reaction cell was fixed under a US-800 solar simulator (150 W Xenon lamp, UNNASOL GmbH, Germany, ~17 mW cm^{-2} incident light power density) equipped with a heat-absorbing filter (HA03, Hebo) and stirred magnetically. Samples were taken at regular intervals, collected in the dark in order to determine the degradation grade of the pollutant, and after finishing the experiment filtered through a micropore filter (Sarstedt, 0.20 μm). The UV/Vis-spectra were recorded with a Cary 60 spectrometer. The amount of total carbon was measured at the Hygiene Institut Gelsenkirchen in a dilution of 1:10. The stability of the photocatalysts was tested during four three-hour cycles of photocatalytic degradation. The catalyst concentration of 2.0 g/L (50 mg) and the 2.4-D concentration of 2.5×10^{-4} M were used. After each cycle the photocatalyst was recovered by centrifugation (4000 rpm, 5 min),

and the 2,4-D concentration was adjusted to the initial value.

The rate of hydroxyl radical generation was determined by measuring the conversion of terephthalic acid (TA) to hydroxyterephthalic acid (TAOH). Photocatalysts were irradiated in TA solution (6×10^{-3} mol/L TA, 0.01 M HCl, pH = 6.5) for 90 min. Samples were collected in 30 min intervals. In the reaction of non-fluorescent TA with hydroxyl radicals, the formation of TAOH can be monitored by emission spectra measurements. TAOH shows a broad emission band at λ_{max} = 425 nm when excited at λ_{exc} = 315 nm. Fluorescence spectra were measured using a FluoroLog-3 (Horiba JobinYvon) spectrofluorometer in a 1 cm quartz cuvette.

Photocatalytic NO_2 conversion was measured in a setup according to the ISO standard 22197-1. All tubing and connections were made of polymers to avoid metal surfaces that could catalytically convert NO_x. NO_2 was supplied by a standard mixture and diluted to about 1 ppm by synthetic air, then delivered at a flow rate of 3 L/min and 50% relative humidity to the reactor. This test gas mixture was passed through the photocatalytic reactor made out of PEEK which comprises a sample holder with the dimensions of 5 × 10 cm^2. In this holder, approximately 2.8 g of the photocatalyst were uniformly dispersed and slightly pressed in with a flat plunger to form a uniform surface. The sample was illuminated from above through a UVA transparent cover glass by a UVA-LED-array (Omicron Laserage Laserprodukte GmbH, Germany) which is calibrated to deliver 365 nm light with 1 mW cm^{-2} intensity at the sample surface. The gas stream is passed above the sample through a 5 mm high slit. The resulting gas stream was analyzed using an environmental NO_x analyzer (Horiba APNA-370).

1.6 Diffuse reflectance spectroscopy

The determination of the electronic absorption properties of the photocatalysts was performed by diffuse reflectance spectroscopy (DRS) using a Perkin Elmer Lambda 650 UV-Vis spectrophotometer with a praying mantis set-up. Each sample (100 mg) was thoroughly ground (0.5 g $BaSO_4$) and loaded into the sample holder. The background reflectance of $BaSO_4$ was used as a reference. The evaluation of the data obtained was conducted according to the Kubelka-Munk function $F(R_\infty)$ as $F(R_\infty) = (1- R_\infty)^2/2R_\infty$, with R_∞ as the diffuse reflectance of the sample relative to the reflectance of a standard ($BaSO_4$).

1.7 X-ray diffractommetry

Powder X-ray diffraction patterns of a-TiO_2, a-TiO_2(400) and a-TiO_2-Cu^{SA} were measured using a Panalytical X´Pert MPD Pro in reflection geometry diffractometer equipped with a Cu K_α source, and samples a-TiO_2-$Fe^{SA,}$ a-TiO_2(400)-V^{SA} and a-TiO2(400)-VO_x^{NP} were analyzed using a STOE STADI-P diffractometer in transmission geometry. X-ray source was Cu $K_{\alpha 1}$ radiation.

1.8 Photoluminescence spectroscopy

Solid-state photoluminescenbce spectra were recorded under excitation wavelength of 330 nm using a Shimadzu RF-6000 spectrometer.

1.9 Raman spectroscopy

Raman spectroscopy was performed on a Renishaw Raman spectrometer using a laser excitation wavelength of 633 nm.

1.10 Transmission electron microscopy (TEM)

The samples were dispersed in ethanol and drop-casted to holey carbon film prior to TEM investigations. Scanning (S) TEM was carried out using a Thermofisher Talos 200X microscope operated at 200 kV. High resolution (HR) and conventional TEM images were acquired using a CETA2 CMOS camera. STEM images were acquired using a high-angle-annular dark-field detector, the microscope was operated in probe mode. Energy dispersive - X-ray spectra were acquired using a windows-less 4 quadrant SuperX dectector parallel with STEM HAADF imaging. Evaluation of the spectra was done using Velox software package (Thermofisher company).

1.11 Inductively coupled plasma optical emission spectrometry (ICP-OES)

For elemental analysis, an ICP-OES (UNICAM 701) was used. 25 mg of each sample was mixed with 800 mg of Na_2O_2 and fused in a Zr-cup over the flame of a Bunsen burner. The melt was diluted in H_2O and HNO_3 and then analyzed with ICP-OES.

1.12 Electron paramagnetic resonance spectroscopy

EPR spectra were recorded at ambient temperature by Bruker X-band ELEXSYS E500 spectrometer operating at 9.7 GHz and 100 kHz magnetic field modulation. In the typical experiments the spectra of transition metal ions doped to the titania were acquired with the microwave power of 2-8 mW, the modulation amplitude of 2-4 G, conversion time 163.84 ms, time constant 81.92 ms, and 4-16 scans were applied. All samples were degassed at room temperature and then activated at 150 °C under vacuum for 30 min prior to the EPR measurements. The samples were UV irradiated for up to 120 min by an ER 203 UV irradiation system (50 W high-pressure mercury lamp, full light). Then the samples were exposed to 50 - 100 Tr of oxygen, 50 Tr methanol vapor, or 50/50 methanol/water mixture at room temperature. To determine the radicals formed during UV irradiation of investigated samples suspension the spin trap techniques was applied. The samples were placed in capillaries, and then the 2% vol DMPO (Sigma-Aldrich) water solutions saturated with oxygen were added and the sample were vigorously shaken. The capillary was placed in the EPR cavity and the system was irradiated by the UV lamp during the registration of the spectra. In the second set of

experiments 1 % vol of methanol water solution, containing 2% vol DMPO was used, and the same procedure was applied. The spectra were simulated by EPRSIM32 software allowing to determine the EPR parameters of all paramagnetic centers centers.[1]

1.13 X-ray absorption measurements (EXAFS/XANES)

Fe and Cu K-edge XAFS spectra were collected at room temperature in a fluorescence mode at XAFCA facility, Singapore Synchrotron Light Source (SSLS). A Bruker Xfalsh 6100 detector was used to measure the fluorescence signal. In a typical experiment, the sample was prepared as a compressed 10 mm diameter pellet and loaded into a cell. The XANES samples were measured 3 times and EXAFS of each sample were measured 10 times to improve the signal to noise ratio of the data. Data were examined by using the XAFS analysis program, Winxas. Fourier transformation of K^3-weighted EXAFS data were performed over the range K = 2 – 8 $Å^{-1}$.

1.14 Photoelectrochemical measurements

To prepare the photoelectrodes, 200 mg photocatalyst were suspended in 1 mL ethanol and sonicated for 15 minutes. Then the suspension was smeared onto the FTO glass by doctor blading using a scotch tape as frame and spacer. The photoelectrodes were dried at 100 °C for 15 minutes and pressed for 2 minutes at a pressure of 200 kg/cm^2. The photoelectrochemical setup consisted of a Gamry 600 Reference potentiostat and a three-electrode cell using a platinum counter electrode and a Ag/AgCl (3 M KCl) reference electrode. The photoelectrodes were pressed against an O-ring of the cell leaving an irradiated area of 0.5 cm^2. The photopotential transient measurements were performed under open circuit conditions. The photoelectrodes were irradiated from the backside (through the FTO glass) by monochromatic light of 350 nm (1.9 mW/cm^2 with FTO) using a Xenon lamp coupled to a monochromator (Instytut Fotonowy, Poland). The measurements were done in a pH 7 phosphate buffer under oxygen atmosphere (normal air) and repeated in an oxygen-free solution (bubbled for 30 minutes with argon). The measurements were repeated with addition of MeOH (1 mL in 40 mL buffer; under air and argon).

1.15 Statistical Analysis

For photocatalytic experiments, standard deviations (σ) were calculated using Excel software from more than three degradation experiments. The error bars were constructed using 2σ values (σ = standard deviation; confidence interval of ~95%).

2. Supplementary figures and notes

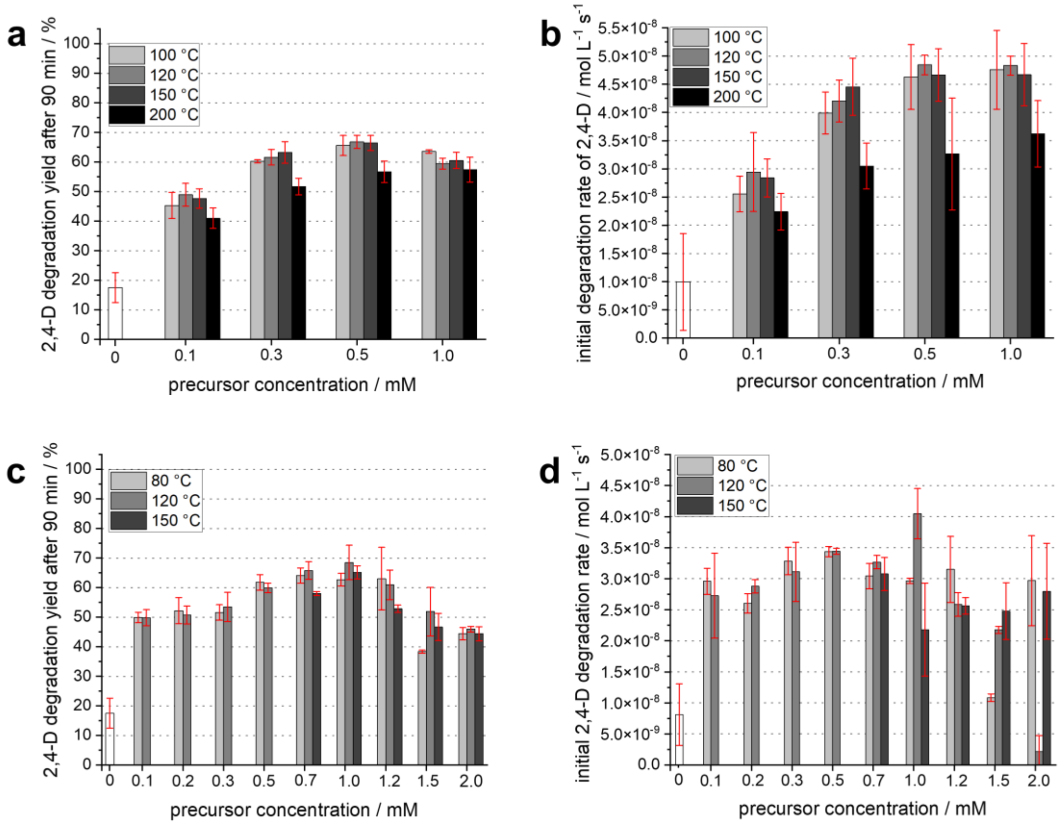

Figure S1. Optimization study of a-TiO_2-Cu and a-TiO_2-Fe samples. Comparison of 2,4-D degradation yields (after 90 min) and initial degradation rates (after 30 min) during photocatalytic degradation experiments using suspensions of pristine anatase TiO_2 (white columns), and a-TiO_2-Cu (a,b) and a-TiO_2-Fe (c,d) prepared under different conditions (precursor concentration, heating temperature). Standard deviations (σ) were calculated from at least three degradation experiments. The error bars were constructed using 2σ values (σ = standard deviation confidence interval of ~95%).

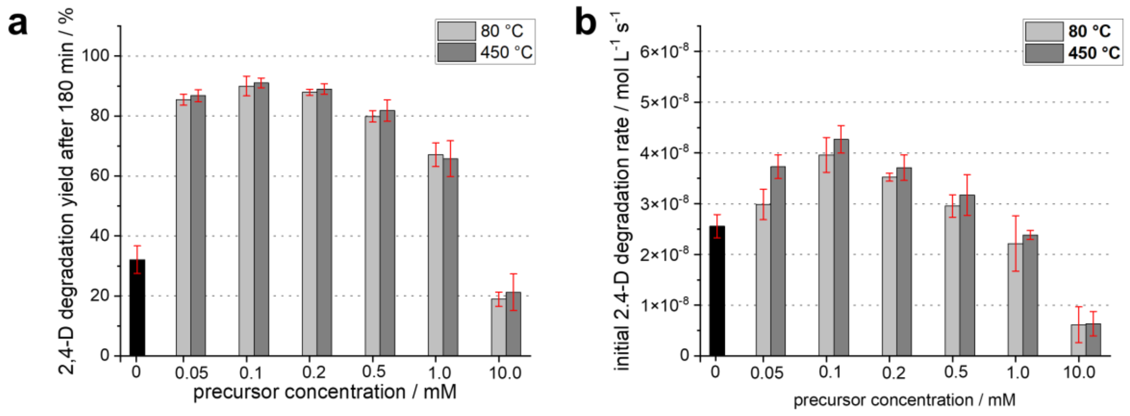

Figure S2. Optimization study of a-TiO_2(400)-V samples. Comparison of 2,4-D degradation yields (a, after 180 min) and initial degradation rates (b, after 60 min) during photocatalytic degradation experiments using suspensions of anatase TiO_2 (black columns), prepared under different conditions (precursor concentration, heating temperature). Standard deviations (σ) were calculated from at least three degradation experiments. The error bars were constructed using 2σ values (σ = standard deviation confidence interval of ~95%).

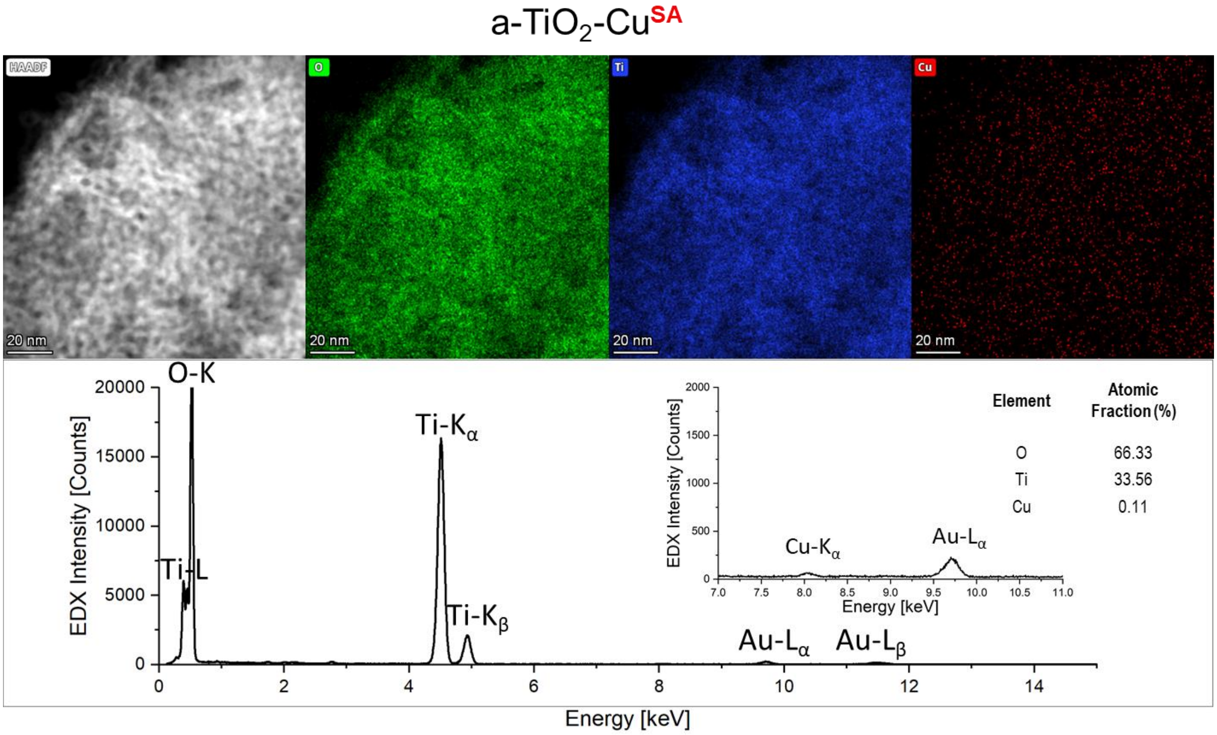

Figure S3. TEM characterization. HAADF image, EDX elemental mappings and integrated EDX spectra of a-TiO_2-Cu^{SA}. (Please note that the Au signals are due to background scattering on the TEM grid.)

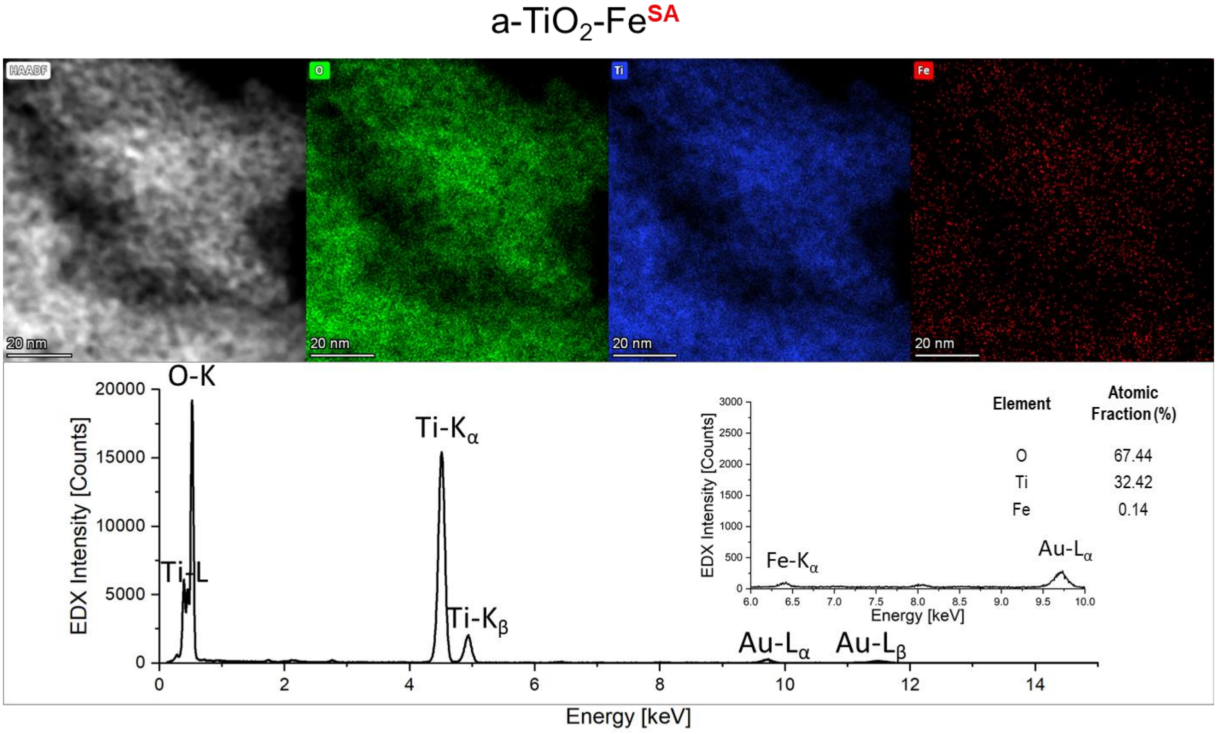

Figure S4. TEM characterization. HAADF image, EDX elemental mappings and integrated EDX spectra of a-TiO_2-Fe^{SA}. (Please note that the Au signals are due to background scattering on the TEM grid.)

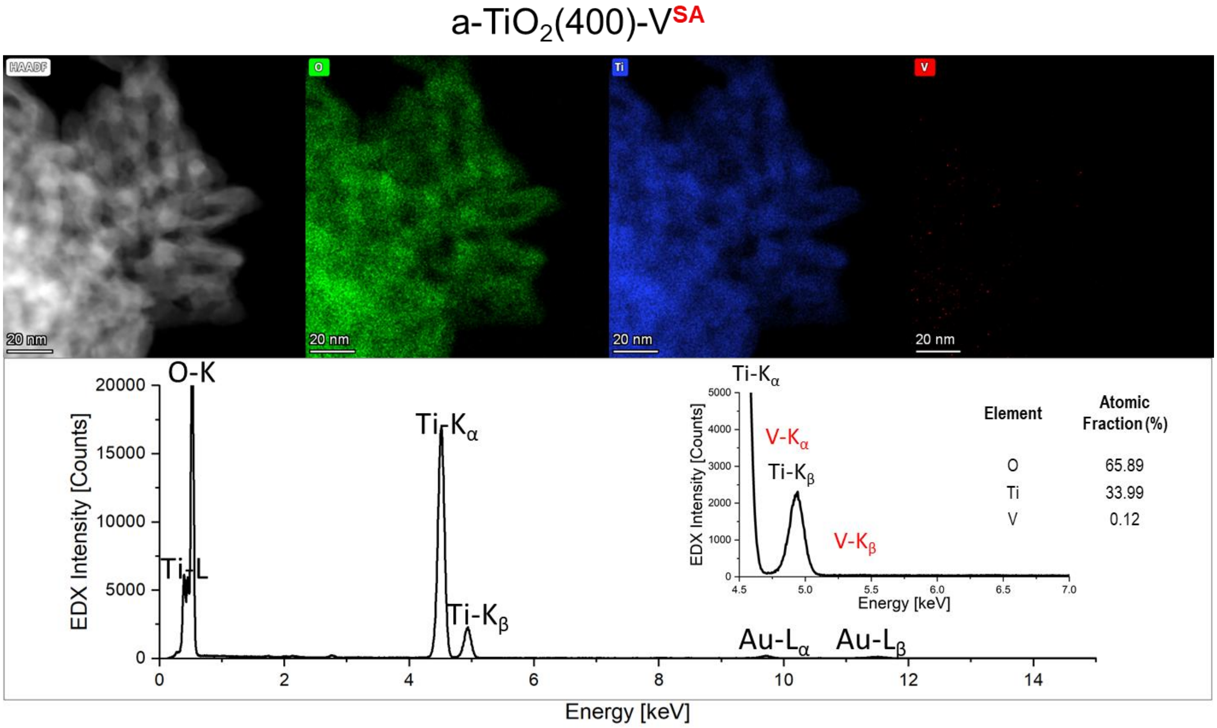

Figure S5. TEM characterization. HAADF image and EDX elemental mapping of a-TiO_2(400)-V^{SA}. Note that the quantification of V content is contentious since the signal of V-K_α overlapped with Ti-K_β, and no signal of V-K_β was visible. (Please note that the Au signals are due to background scattering on the TEM grid.)

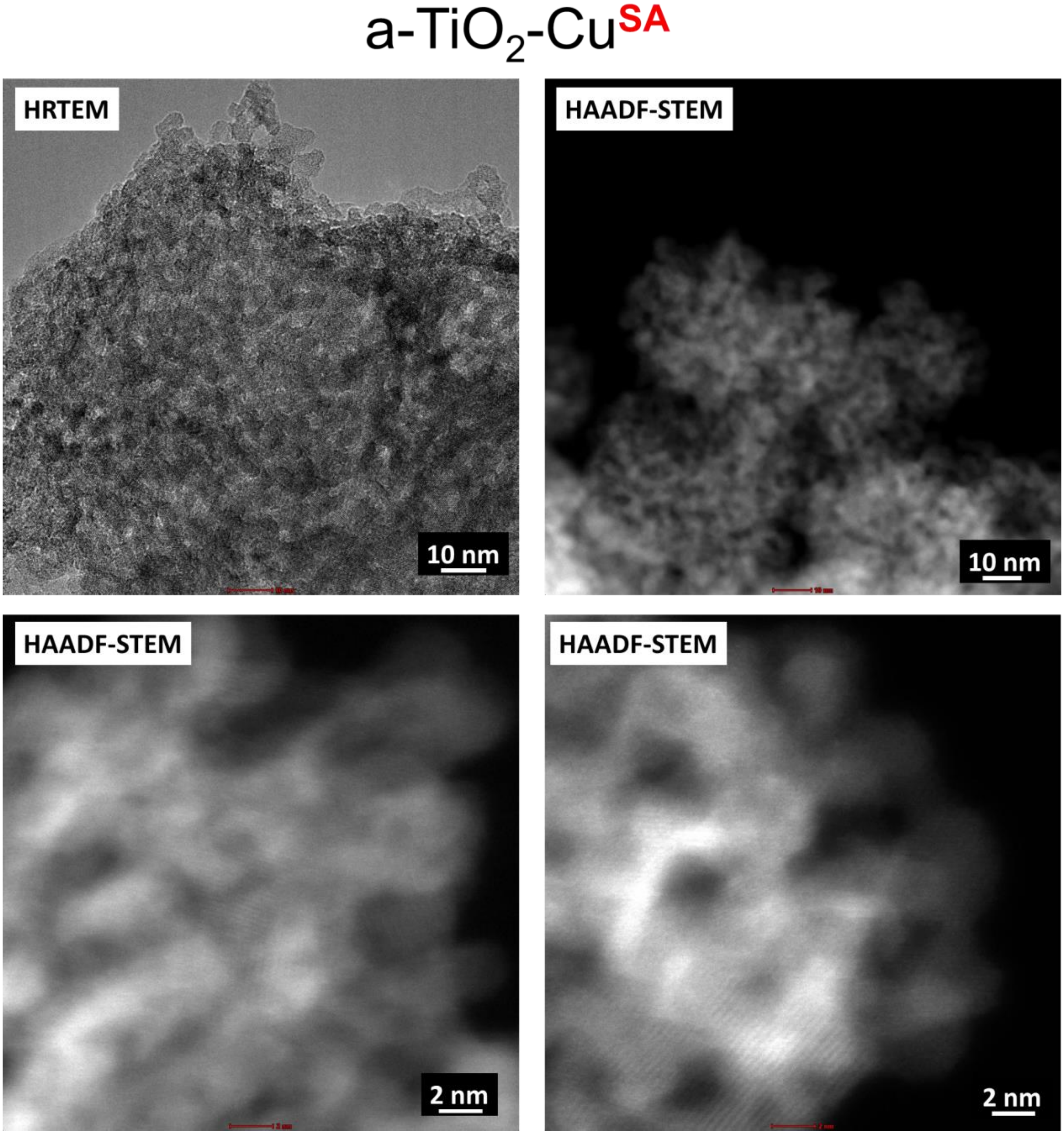

Figure S6. TEM characterization. HRTEM and HAADF-STEM images of a-TiO_2-Cu^{SA}. Direct imaging of single Cu(II) ions within the TiO_2 substrate was impossible due to the low amount of Cu, and the very low Z-contrast difference with respect to Ti.

$a\text{-}TiO_2\text{-}Fe^{SA}$

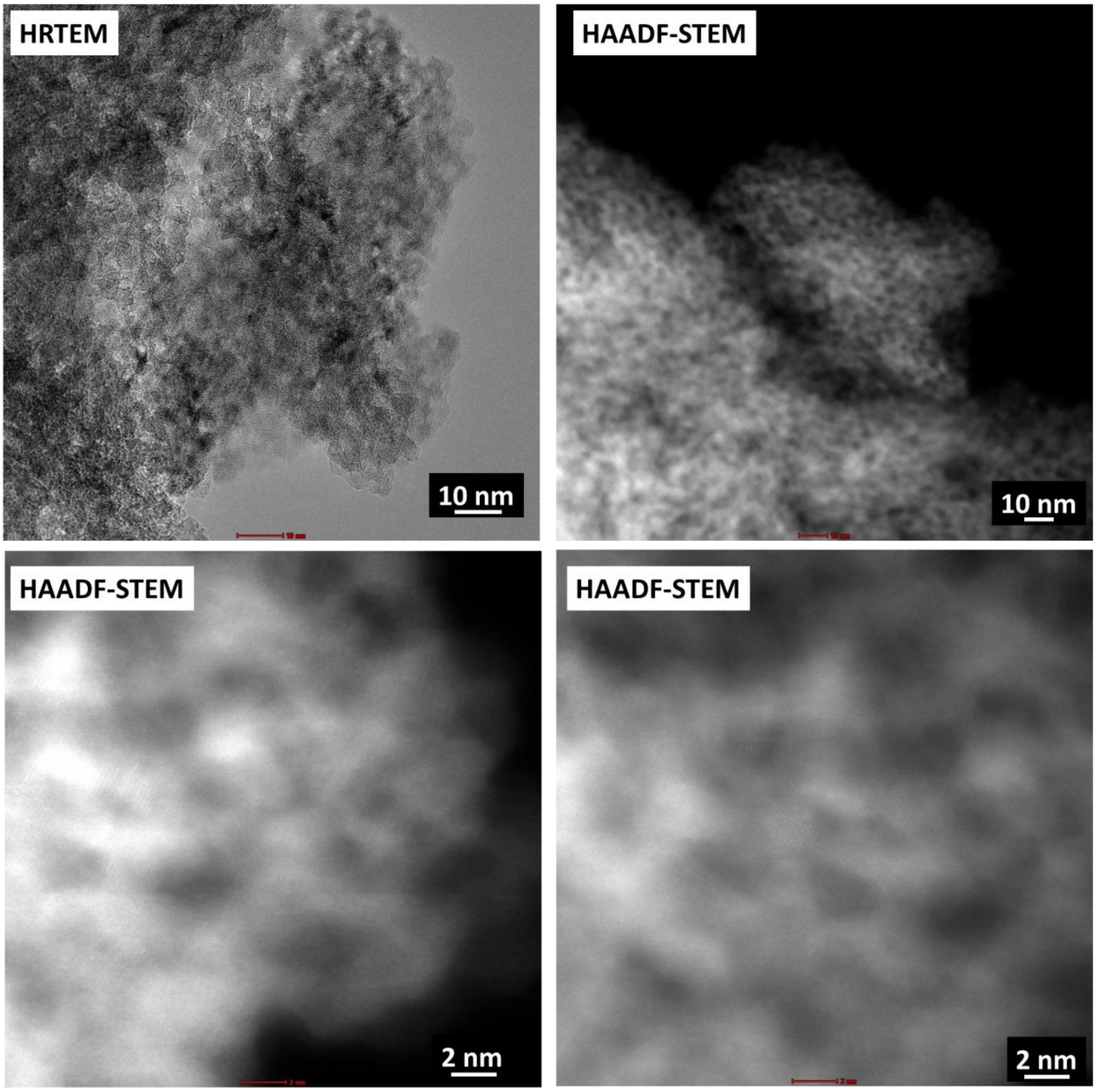

Figure S7. TEM characterization. HRTEM and HAADF-STEM images of $a\text{-}TiO_2\text{-}Fe^{SA}$. Direct imaging of single Fe(III) ions within the TiO_2 substrate was impossible due to the low amount of Fe, and the very low Z-contrast difference with respect to Ti.

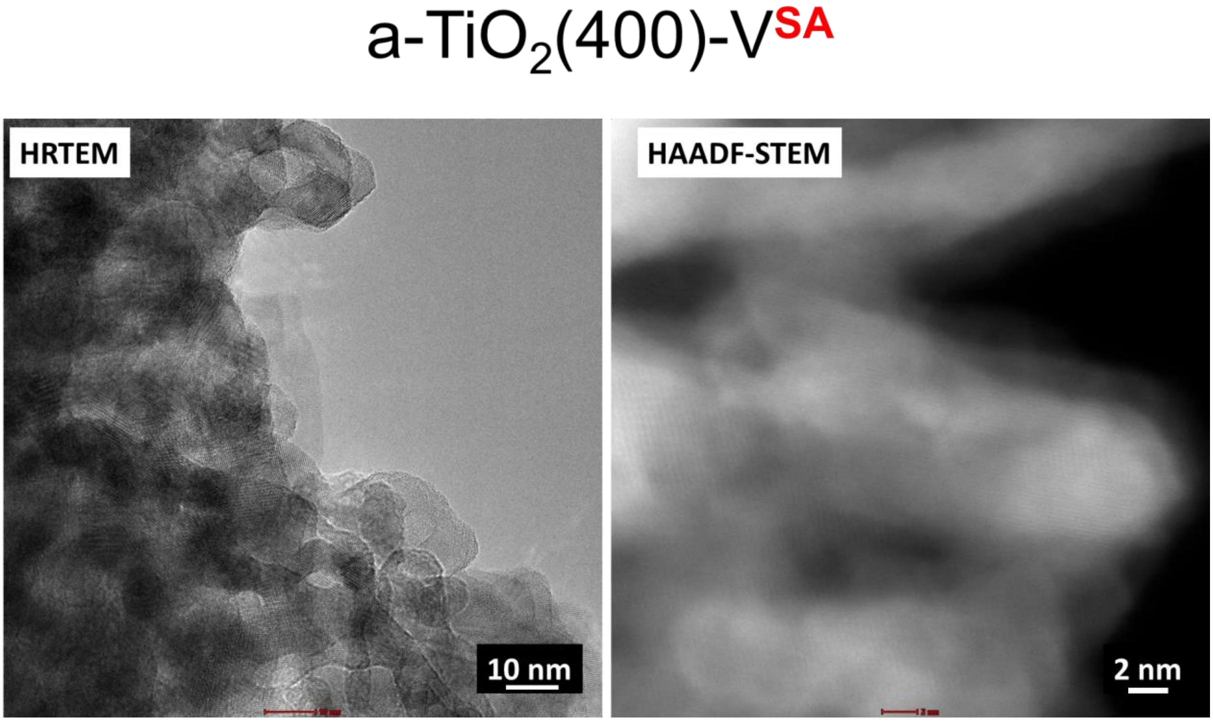

Figure S8. TEM characterization. HRTEM and HAADF-STEM images of a-TiO_2(400)-V^{SA}. Direct imaging of single vanadate ions within the TiO_2 substrate was impossible due to the low amount of vanadate, and the very low Z-contrast difference with respect to Ti.

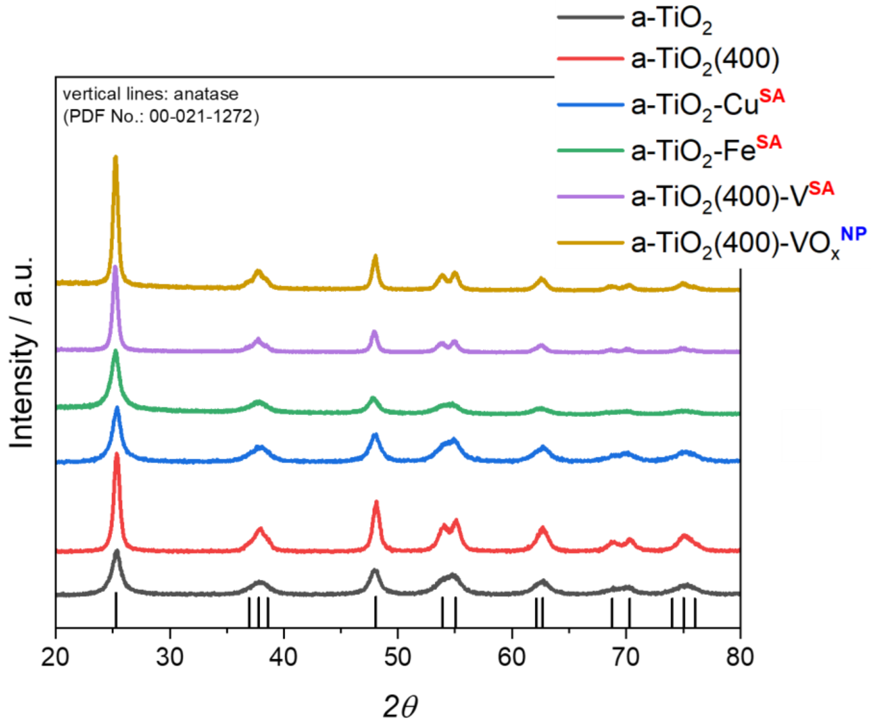

Figure S9. Powder X-Ray diffractommetry. For all samples only reflexes from anatase TiO_2 were detected.

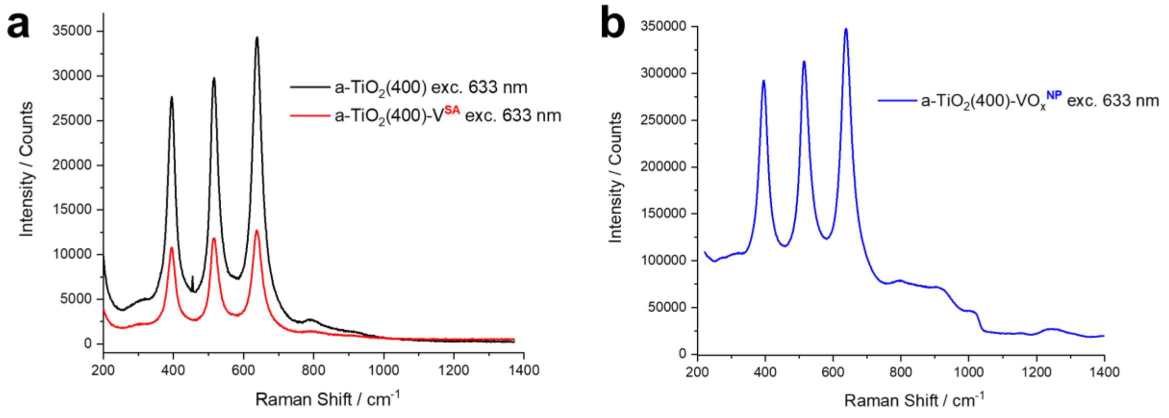

Figure S10. Raman spectroscopy. Raman spectra of the unmodified sample a-TiO_2(400) and the vanadate SAC-modified sample a-TiO_2(400)-V^{SA} show only peaks from the anatase TiO_2 phase (a). In contrast, the Raman spectrum of the TiO_2 sample modified with VO_x nanoparticles (a-TiO_2(400)-VOx^{NP}) reveals also additional vibrational contributions that can be ascribed to metavanadate $(VO_3)_n$ (~940 cm^{-1}) and V_2O_5 (~994 cm^{-1}).[2]

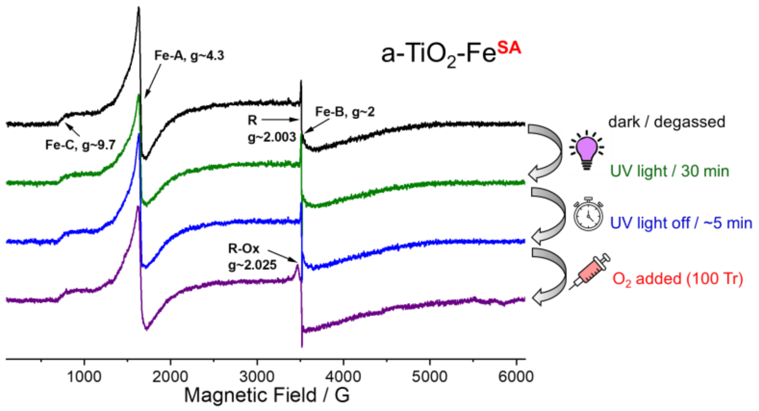

Figure S11. EPR spectroscopic analysis of a-TiO_2-Fe^{SA}. *In-situ* X-Band EPR spectrum of degassed a-TiO_2-Fe^{SA} sample (black) in the dark, after UV-irradiation for 30 min (green), after 5 min after switching off the UV light (blue), and after interaction with **100** Tr of oxygen (magenta).

Note 1: EPR analysis of a-TiO_2-Fe^{SA}

In Figure S11, the EPR spectra of sample a-TiO_2-Fe^{SA} are presented. The main signal (Fe-A) with effective g_{eff} factor around ~4.3 originated from transition within the middle Kramer's doublet of the high spin iron complex with S=5/2 and was attributed to isolated Fe(III) ions at the surface or near surface of TiO_2 ,[3]. Additionally, not resolved resonances are observed in the range of g factor between 5 and 9. The signals with g around 6 are the perpendicular components of anisotropic signal with no visible parallel g line and appeared due to transition within the lowest Kramer doublet and can be qualified as penta- or hexacoordinated Fe(III) ions exhibiting octahedral with strong tetrahedral distortion symmetry. The line with the highest

g value (g_{eff}~9.7, only one visible in this range of magnetic field) was assigned to another type of distortion of the originally tetrahedral Fe(III) centers. [4]. In the case of low spin Fe(III) complexes, with S=1/2, the expected transition is characterized by g around 2 and a relatively sharp line. We cannot rule out the presence of Fe(II) because, usually such species are not visible in X-band at room temperature.[3c] Radicals like signal with g~2.003 (R)`, alike to the a-TiO_2-Cu^{SA}, was assigned to the TiO_2 defects. The broad signal around g~2 (Fe-B) might be assigned to small iron oxide-type clusters wrapped up by the anatase matrix.[5] The EPR signal intensity slightly decreases upon UV irradiation whereas interaction with oxygen at ambient temperature does not have significant effects on the signal originating from iron species. However, after the addition of dioxygen, a new signal appear (R-Ox) characterized by $g_z \cong$ 2.025, $g_y \cong$ 2.023, $g_x \cong$ 2.006 and with the shape typical for oxygen originated radicals.[6]

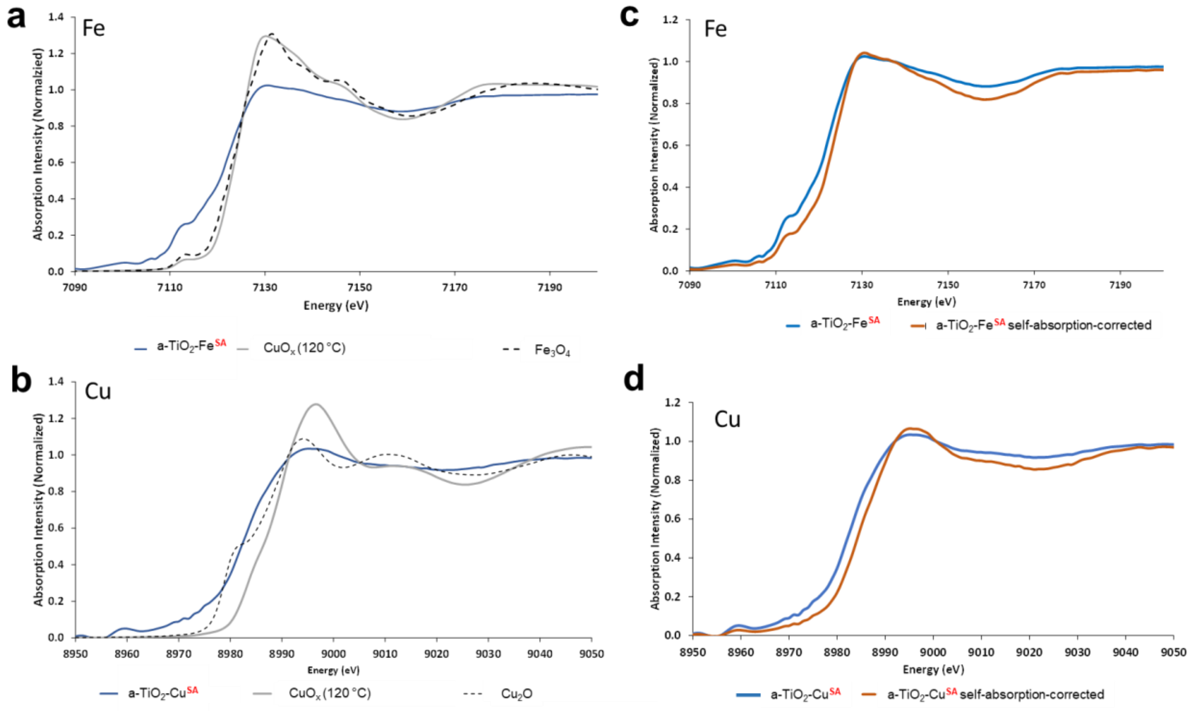

Figure S12. X-ray absorption spectroscopy. X-ray Absorption Near Edge Structure (XANES) data of both the a-TiO_2-Fe^{SA} (a) and a-TiO_2-Cu^{SA} (b) photocatalysts. Data is compared to reference standards. Reference CuO_x and FeO_x samples were prepared by precipitation of copper and iron hydroxides from nitrate solutions, followed by filtration and drying at 120 °C. Data in (c) and (d) show XANES data for a-TiO_2-Fe^{SA} and a-TiO_2-Cu^{SA} at the Fe and Cu edges, respectively, with self-absorption corrections (red indicates the corrected data). Self-absorption corrections are estimates based on a 0.2 at.% metal content in an anatase matrix, and are calculated using the Athena.

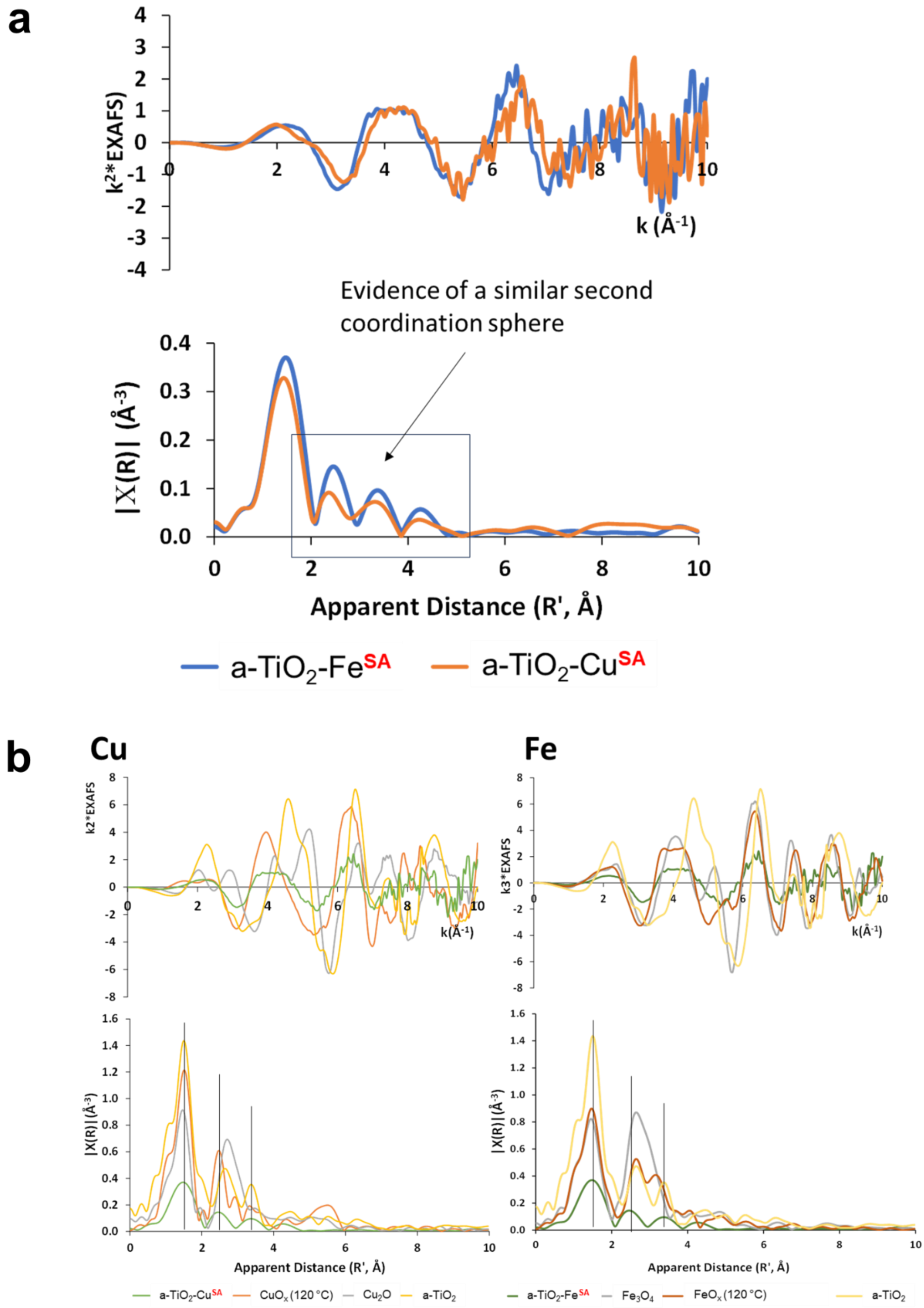

Figure S13. X-ray absorption spectroscopy. (a) Comparison of EXAFS and Fourier transform of the EXAFS for a-TiO_2-Fe^{SA} and a-TiO_2-Cu^{SA}. (b) Comparison of EXAFS and Fourier transform of the EXAFS of a-TiO_2-Fe^{SA} and a-TiO_2-Cu^{SA} to reference EXAFS spectra of anatase TiO_2 (Ti K edge) and various reference copper and iron oxides.

Note 2: X-ray absorption analysis

XANES data for both TiO_2-Fe^{SA} and a-TiO_2-Cu^{SA} is given in Figure S12a,b compared to reference standards. The Fe data is shifted to lower energy relative to the Fe(III) reference standards. Similarly the copper data is shifted to an energy more similar to the Cu(I) reference Cu_2O rather than the Cu(II) materials. However, while XANES is frequently used to examine the oxidation state, in our materials the XANES position is affected by the sample matrix. To give an indication of how the TiO_2 matrix effects the XANES position, self-absorption corrections[7] were performed in Athena, and are presented in Figure S12ac,d . Given the nature of these materials, *i.e.* Cu/Fe (~0.2 at%) supported on TiO_2, the self-absorption correction to account for matrix effects is difficult and the resolution of the XANES is therefore limited. Given the clear EPR evidence for Cu(II) in a-TiO_2-Cu^{SA}, we conclude that the a-TiO_2-Cu^{SA} system contains mainly Cu(II), though it cannot be ruled out that very small amount of EPR-silent Cu(I) is present. Similarly, given the complications by the TiO_2 matrix, the XAS data are consistent with conclusions from the EPR analysis that show the presence of Fe(III) in a-TiO_2-Fe^{SA}.

To understand the structure of the materials, an EXAFS analysis was undertaken at both the Fe/Cu and Ti edge. Fundamentally, when considering the nature of Cu(II) and Fe(III) species on TiO_2 , three extreme scenarios can be proposed. Either **i)** the Cu and Fe species can act as a substitutional dopant, or **ii)** they can form a surface (physi)sorbed species, or **iii)** they can form a composite of TiO_2 and CuO_x/FeO_x. These differences in boding can be separated by X-ray Absorption spectroscopy and analysis of the EXAFS region.[7] Figure S13a compares the a-TiO_2-Cu^{SA} material to the a-TiO_2-Fe^{SA} material both in the EXAFS and the Fourier transform of the EXAFS. Interestingly, the two materials are very similar in that both have a well-defined second coordination sphere that is above error. This rules out the presence of only loosely surface-physisorbed species as being a major component since in such a case the materials would not typically have a well-defined second coordination sphere. To estimate the possibility of scenarios **i**) and **iii)**, the materials were compared to references in Figure S13b. If Cu and Fe were acting as substitutional dopants in anatase TiO_2, then the second coordination sphere and EXAFS at the Cu and Fe edge would be identical to anatase (Ti K edge), which is not the case, nor is it, however, the same for any of the expected Cu and Fe references, which rules out the presence of CuO_x and FeO_x clusters. Thus, given the similarity of the second sphere (Figure S13a), we believe the bonding scenario in our a-TiO_2-Cu^{SA} and a-TiO_2-Fe^{SA} materials is best understood as a middle case between the scenario **i)** and **ii)**, i.e. between a substitutional dopant and only physisorbed ions. In other words, the Cu(II) and Fe(III) ions are strongly bound, rather than weakly physisorbed, to the anatase TiO_2 surface.

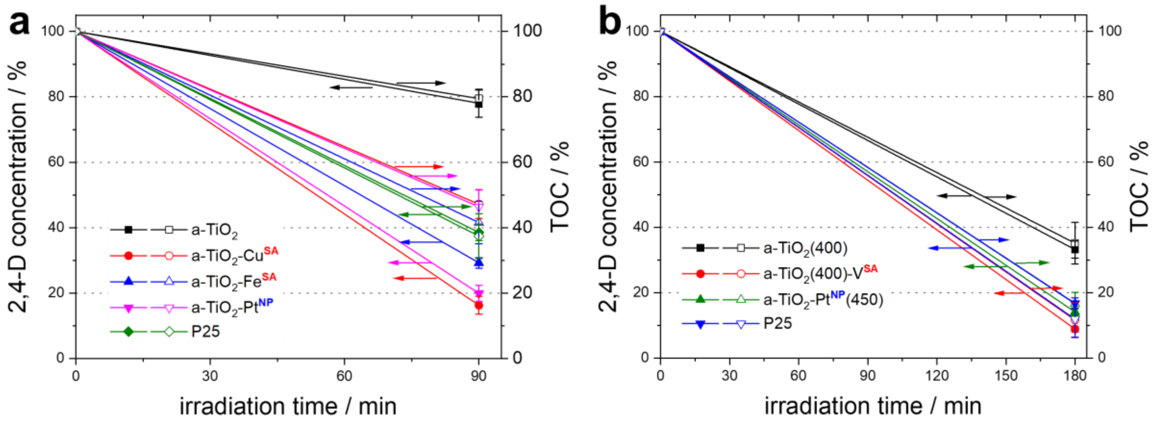

Figure S14. Evidence for mineralization of the 2,4-D pollutant during photocatalysis. Comparison of the 2,4-D concentrations (determined using UV-Vis absorption spectroscopy) and total organic carbon (TOC) values during photodegradation experiments.

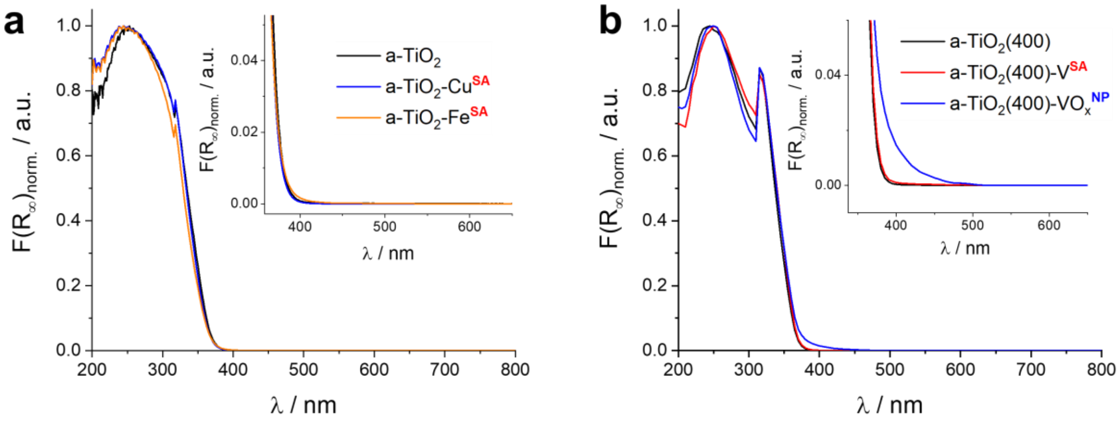

Figure S15. Electronic absorption properties of studied photocatalysts. Normalized diffuse reflectance spectra (Kubelka-Munk function *vs*. wavelength) of anatase TiO_2 materials modified with Cu(II), Fe(III) (a) and with vanadate SACs (b). In (b) note the light absorption extended to the visible range (down to > 500 nm) for the reference sample modified with VO_x nanoparticles (a-TiO_2(400)-VO_x^{NP}), which is in stark contrast to the light absorption properties of isolated single vanadate species, which are known to absorb only under 320 nm,[8] and the electronic absorption of a-TiO_2(400)-V^{SA} is therefore expected to be dominated by the optical properties of anatase TiO_2.

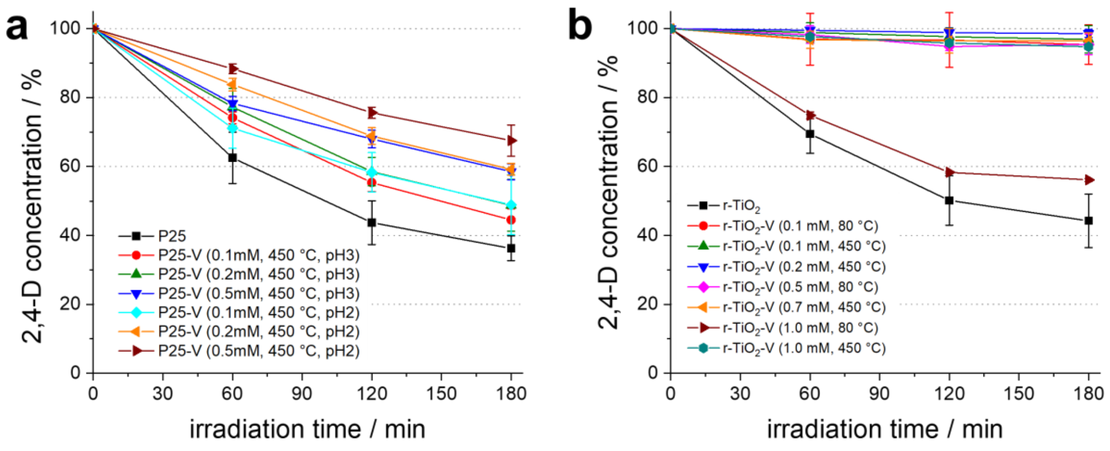

Figure S16. Photocatalytic activity of vanadate-modified P25 (a) and rutile TiO_2 (b) substrates. The concentrations and pH values in the parentheses designate the concentration and pH of the precursor solution during impregnation, the temperature designates the heat treatment temperature. The actual vanadium contents of selected photocatalysts are listed in **Table S1** below.

Table S1. Vanadium content of different samples based on P25 and rutile TiO_2 measured by ICP-OES.

**sample**	**V [wt%]**
P25-V (0.1 mM, pH3)	0.03
P25-V (0.2 mM, pH3)	0.04
P25-V (0.5 mM, pH3)	0.08
r-TiO_2-V (0.1 mM, pH2)	0.03
r-TiO_2(R)-V (0.1 mM, pH3)	0.03
r-TiO_2(R)-V (0.2 mM, pH2)	0.04
r-TiO_2(R)-V (0.2 mM, pH3)	0.03

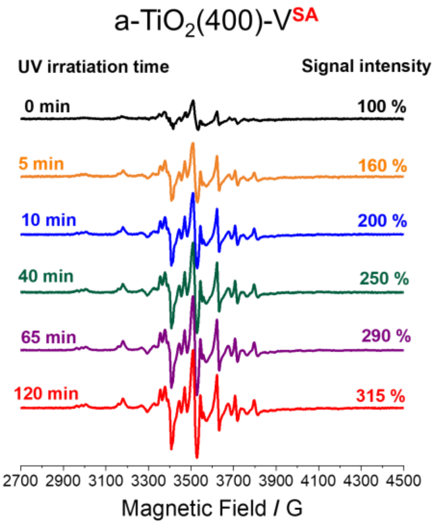

Figure S17. EPR spectroscopic analysis of a-TiO_2(400)-V^{SA}. Time-resolved signal development during the initial photoreduction under UV irradiation in the absence of oxygen.

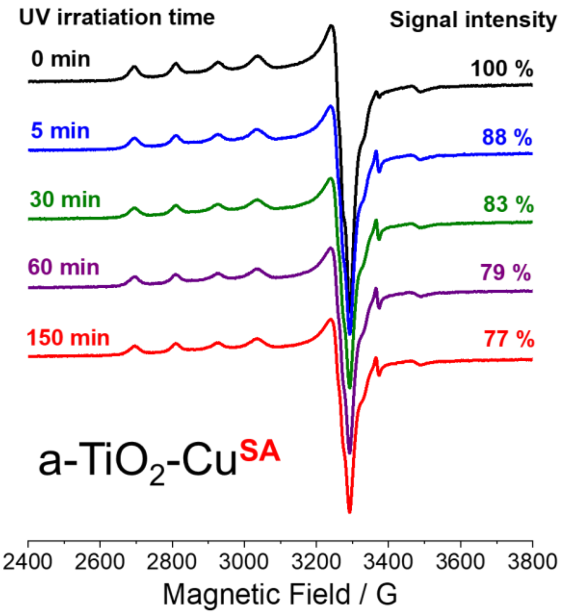

Figure S18. *In-situ* X-Band EPR spectroscopy of a-TiO_2-Cu^{SA}. Time-resolved development of the EPR signal upon UV light-induced reduction in the *absence* of methanol vapor.

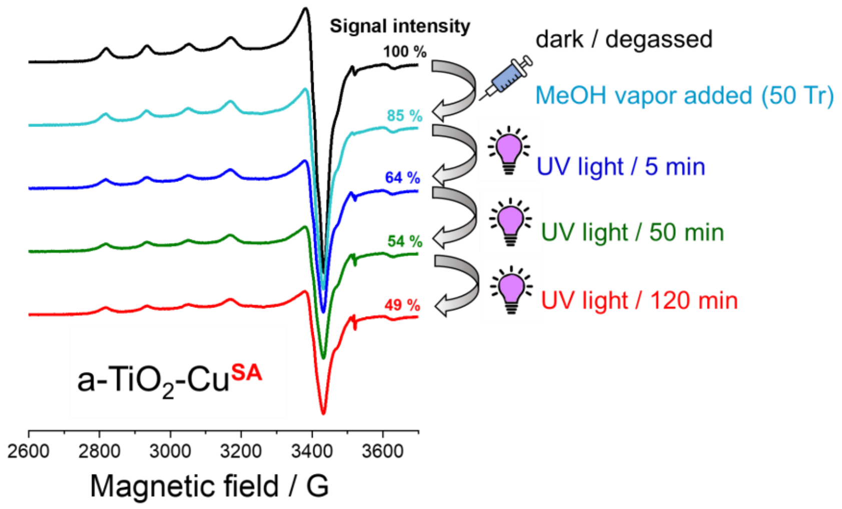

Figure S19. ***In-situ*** **X-Band EPR spectroscopy of a-TiO₂-Cu^{SA}.** Time-resolved development of the EPR signal upon UV light-induced reduction in the *presence* of methanol vapor.

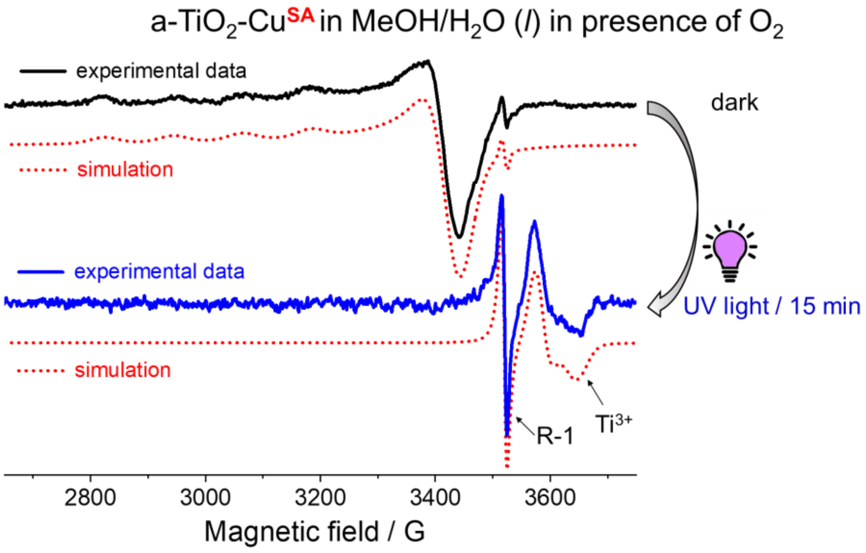

Figure S20. ***In-situ*** **X-Band EPR spectroscopy of a-TiO₂-Cu^{SA}**. EPR signal of single Cu(II) sites before and after UV light-induced reduction in a liquid water/methanol (50/50 vol.) mixture containing oxygen accompanied by spectra simulation (red).

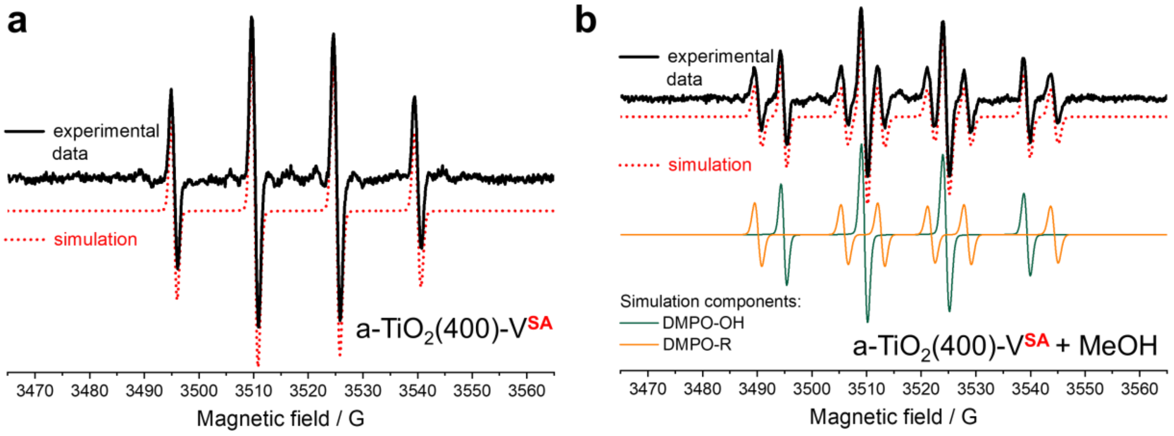

Figure S21. *In-situ* X-Band EPR spectroscopy of detected radicals. Experimental (solid black line) and simulated (dotted red line) X-Band EPR spectra of DMPO adducts revealed in (a) water and (b) water methanol (99/1) suspensions of a-TiO_2(400)-V^{SA} containing 2 vol% of spin trap (the simulated components are given at the bottom) recorded upon UV irradiation. All samples, either unmodified or SAC-modified, exhibited nearly identical spectra. The results for all samples are summarized in **Table S2** below. For details see the **Note 3** below.

Table S2. The radicals detected upon UV irradiation of photocatalyst suspensions.

Sample	Detected radicals	
	**Water – 2 vol% DMPO**	**Water – 2 vol% DMPO 1 vol% MeOH**
a-TiO_2	•OH	•OH and •R (carbon centered radicals)
a-TiO_2(400)	•OH	•OH and •R (carbon centered radicals)
a-TiO_2(400)-V^{SA}	•OH	•OH and •R (carbon centered radicals)
a-TiO_2-Cu^{SA}	•OH	•OH and •R (carbon centered radicals)
a-TiO_2-Fe^{SA}	•OH	•OH and •R (carbon centered radicals)

Note 3: *In-situ* X-Band EPR spectroscopy of detected radicals

In order to detect the radicals generated by investigated photocatalysts upon UV irradiation, EPR spin trap techniques were applied and experiments with sample suspensions in water and water/methanol mixture were performed. In Figure S21a the EPR spectra of DMPO adducts detected in a-TiO_2(400)-V^{SA} suspension in water are presented. The signal shown in Figure S21a consists of four lines with the relative intensities 1:2:2:1 and magnetic parameters g = 2.0053, A_N = 14.9 G A_H = 14.7 G as determined by spectrum simulation**.** This signal is typical for DMPO adducts with hydroxyl radicals, and was denoted DMPO–OH.[9]

In the case of a-TiO_2(400)-V^{SA} suspension in water/methanol mixture additional signal with six lines was observed (Figure S21b), and simulated with following parameters g = 2.0053, A_N = 15.8 G A_H = 22.5 G. The signal shape and parameters are typical for the DPO adduct with carbon centered radicals, and the one was denoted as DMPO–R. These radicals were created by the attack of hydroxyl radicals on methanol present in the solution. The similar testing were done for all catalysts (**Table S2**) and the same results were obtained. In performed experiments superoxide anion radicals were not detected, possibly because DMPO–OOH adducts are unstable and spontaneously decompose into a nonradical species and DMPO–OH.[10]

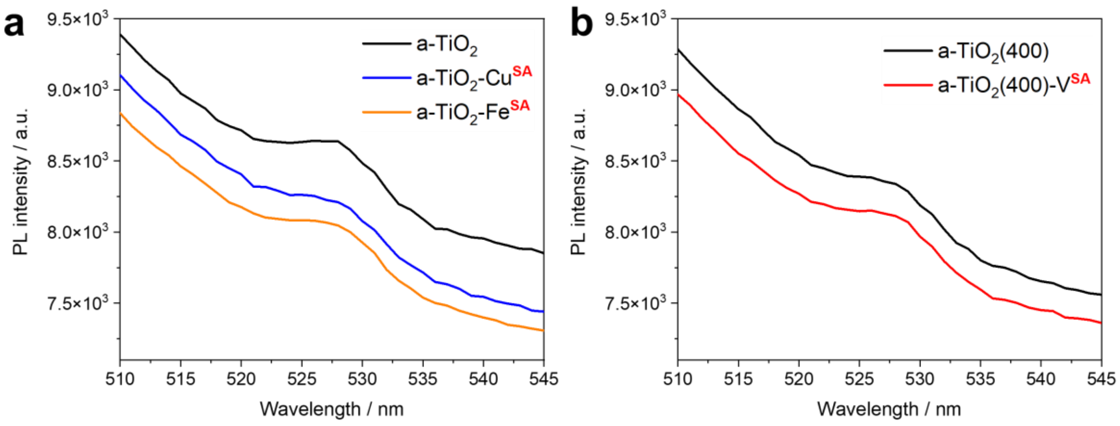

Figure S22. Quenching of solid-state photoluminescence. Solid-state photoluminescence spectra of a-TiO_2-CuSA, a-TiO_2-FeSA (a) and a-TiO_2(400)-VSA (b) with respect to the PL spectra of unmodified pristine samples a-TiO_2 (a) and a-TiO_2(400) (b). The photoluminescence band centered in the green region (~2.35 eV, ~528 nm) of the optical spectrum, ascribed to the radiative recombination of conduction band electrons with trapped holes,[11] is partially quenched in the presence of SACs, which confirms the beneficial effect of SACs on the electron extraction from TiO_2. The excitation light wavelength was 330 nm. We note that the same trends have been observed also at the excitation wavelength of 360 nm.

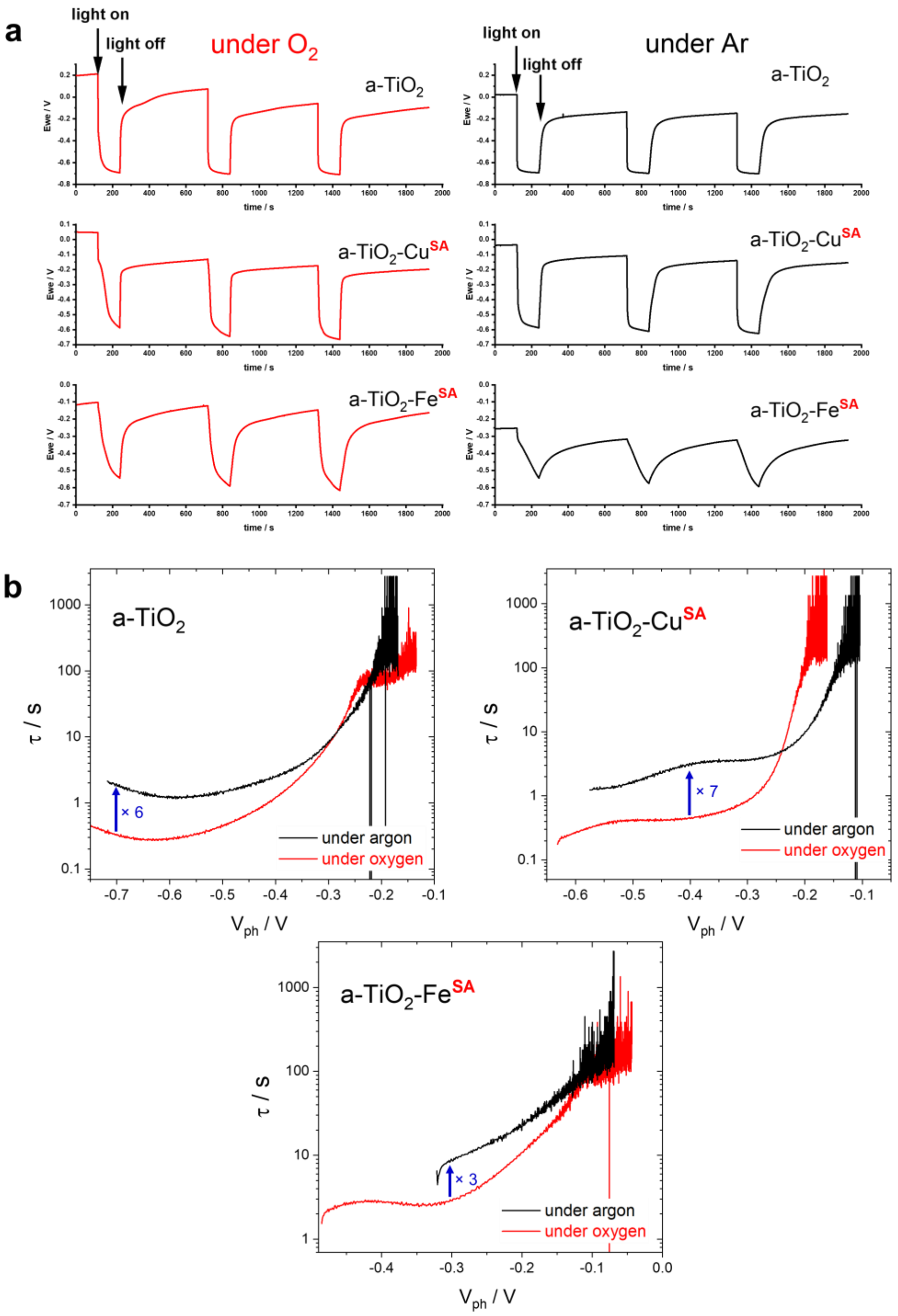

Figure S23. The effect of SACs on photogenerated electron lifetime. (a) Open-circuit potential transients recorded under interrupted monochromatic illumination (λ = 350 nm) for pristine a-TiO_2, a-TiO_2-Cu^{SA}, and a-TiO_2-Fe^{SA} deposited on FTO glass. The transients were recorded in methanol-containing (2.5 vol%) 0.1 M phosphate buffer (pH 7) under oxygen atmosphere (normal air) and in an oxygen-free environment (bubbling with argon for 30 min). The electrodes were irradiated from the backside. (b) Electron lifetime τ as a function of photopotential V_{ph} for different samples derived from photopotential decay transients after switching-off the light (using Equation 3 in **Note 4** below). The numbers next to the arrows indicate the relative decrease of electron lifetime in the presence of oxygen *versus* without oxygen. The data shown were derived from the second transient decay (a); the lifetime trends were practically the same for all transients.

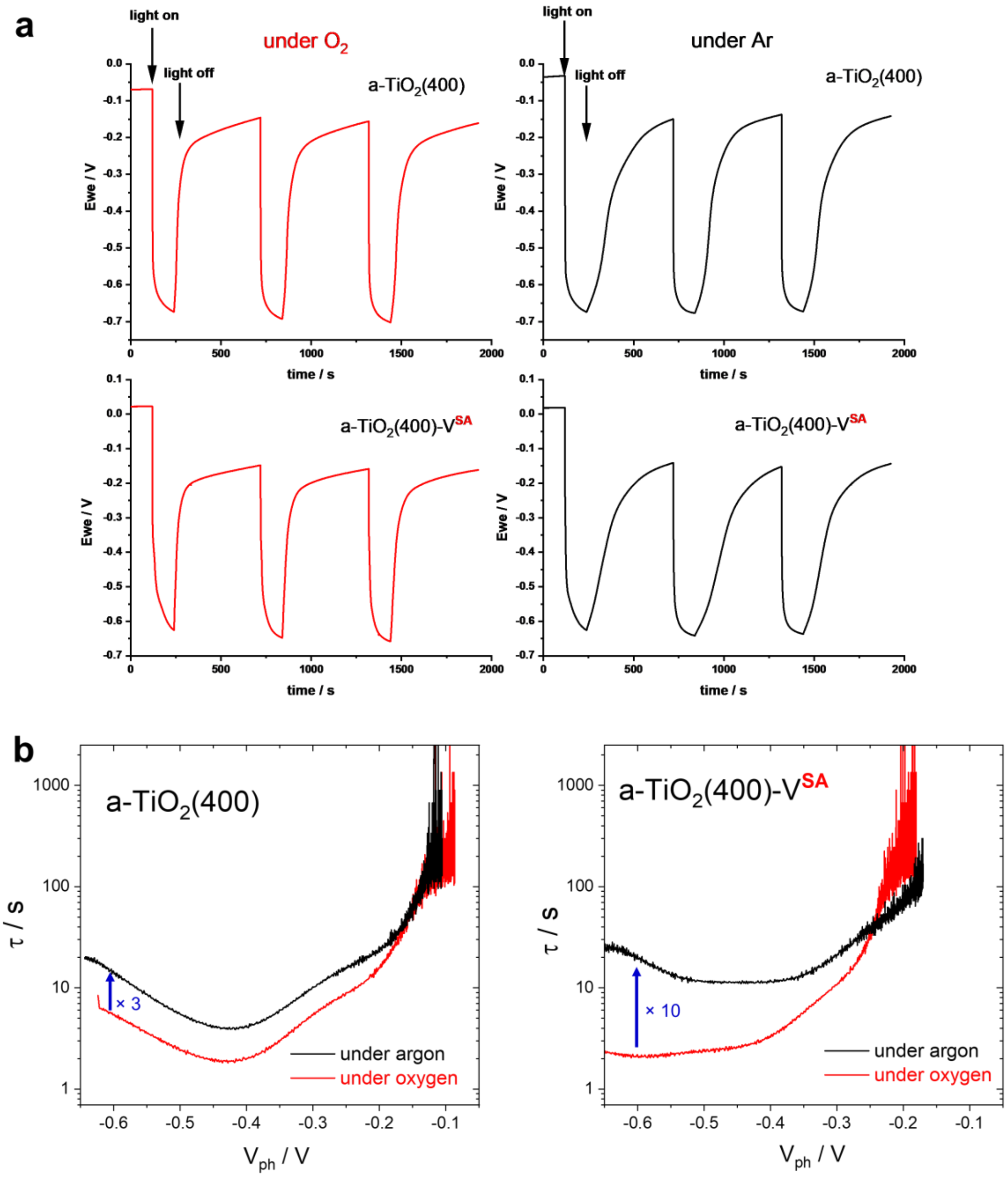

Figure S24. The effect of SACs on photogenerated electron lifetime. (a) Open-circuit potential transients recorded under interrupted monochromatic illumination (λ = 350 nm) for pristine a-TiO_2(400) and vanadate-modified a-TiO_2(400)-V^{SA}. The transients were recorded in methanol-containing (2.5 vol%) 0.1 M phosphate buffer (pH 7) under oxygen atmosphere (normal air) and in an oxygen-free environment (bubbling with argon for 30 min). The electrodes were irradiated from the backside. (b) Electron lifetime τ as a function of photopotential V_{ph} for different samples derived from photopotential decay transients after switching-off the light (using Equation 3 in **Note 4** below). The numbers next to the arrows indicate the relative decrease of electron lifetime in the presence of oxygen *versus* without oxygen. The data shown were derived from the second transient decay (a); the lifetime trends were practically the same for all transients.

Note 4: Photopotential transients and electron lifetime determination:

As expected, TiO_2-based photoelectrodes show negative photopotentials since, during irradiation, photogenerated electrons accumulate in TiO_2 and are transported by drift-diffusion to the underlying FTO conductive glass electrode, shifting thus its Fermi level to more negative electrode potentials (Figs. S23a and S24a). After switching-off the light, the photopotential decays principally due to two processes: i) recombination, and ii) interfacial electron transfer to species in the electrolyte. The differences in kinetics that are related distinctly to the differences in the rate of reaction with oxygen are thus accessible when decay kinetics are compared between measurements done in the absence and in the presence of dissolved oxygen. In order to account at least semi-quantitatively for the different reaction rates with oxygen at TiO_2 materials without and with different SACs, we follow the simplified formalism of Zaban *et al.*,[12] and derive the momentous electron lifetime in dependence on the momentous photopotential.[13] Assuming the first-order kinetics of global electron consumption (by both recombination and electron transfer) with respect to the concentration of electrons (n) in the TiO_2 porous electrode, the electron lifetime τ can be defined as $\tau = -\frac{1}{n}\frac{dn}{dt}$ (1). The measured photopotential V_{ph} is defined as the difference in open-circuit potential under illumination and in the dark, $V_{ph} = V_{oc}^{light} - V_{oc}^{dark}$. Using the Boltzmannian approximation of the Fermi-Dirac distribution function (*i.e.*, assuming $^*E_{Fn} - E_c$ □ kT, where E_c is the energy of the conduction band edge and $^*E_{Fn}$ is the *quasi*-Fermi level of electrons), V_{ph} can be expressed as $V_{ph} = -\frac{kT}{e}\ln\left(\frac{n}{n_0}\right)$ (2), where e is the (positive) elementary charge, k is the Boltzmann constant, T is the absolute temperature, and n_0 is the concentration of electrons in the dark. By combining equations (1) and (2), we obtain for the electron lifetime the relation: $\tau = \frac{kT}{e}\left(\frac{dV_{ph}}{dt}\right)^{-1}$ (3).

Figures S23b and S24b show that, for all samples, the electron lifetimes at sufficiently negative photovoltages (*i.e.*, at relatively high concentrations of photogenerated electrons accumulated in TiO_2) are systematically lower in oxygen-containing solutions than in the absence of oxygen. This is expected since an additional electron consumption pathway, apart from recombination, is made available by the presence of oxygen as an electron acceptor. When estimating the activity of a single-atom co-catalyst in enhancing the rate of dioxygen reduction, an important figure of merit is the factor by which the electron lifetime is shorter in the presence of oxygen as compared to the situation in the absence of oxygen. Since different samples, in general, exhibit photovoltages in a different range, we compare the factors estimated at the

photovoltage where the difference in electron lifetimes obtained in the presence and absence of dioxygen is maximal.

Most importantly, and fully in line with our mechanistic investigations by EPR spectroscopy, we observe a significant difference in the behaviour of the samples a-TiO_2-Cu^{SA} and a-TiO_2-Fe^{SA} on the one side, and the sample a-TiO_2(400)-V^{SA} on the other side. At a-TiO_2-Cu^{SA} and a-TiO_2-Fe^{SA} the electron lifetime in the presence of dioxygen is shorter by the factor of 7 and 3, respectively (Figure S23b), which is not significantly higher than in case of the unmodified a-TiO_2 (factor of 6). These results suggest that at both a-TiO_2-Cu^{SA} and a-TiO_2-Fe^{SA} the kinetics of the primary reduction of O_2 by photogenerated electrons is not significantly enhanced as compared to pristine a-TiO_2. In contrast, for the sample a-TiO_2(400)-V^{SA} we observe that the electron lifetime in the presence of oxygen is shorter by the factor of 10, which is significantly higher than in case of the corresponding pristine material a-TiO_2(400) where the factor is only 3 (Figure S24b). These results suggest that in the presence of vanadate SAC, the rate of dioxygen reduction by photogenerated electrons is truly enhanced as compared to the situation without the SAC catalyst. The results of electron lifetime measurements thus corroborate strongly our conclusion about the fundamental difference in the catalytic enhancement of dioxygen reduction at photocatalysts modified with vanadate SACs as compared to Cu(II) SACs, and indicate that the case of Fe(III) SACs is rather similar to Cu(II), *i.e.*, the rate of primary O_2 reduction is not significantly enhanced.

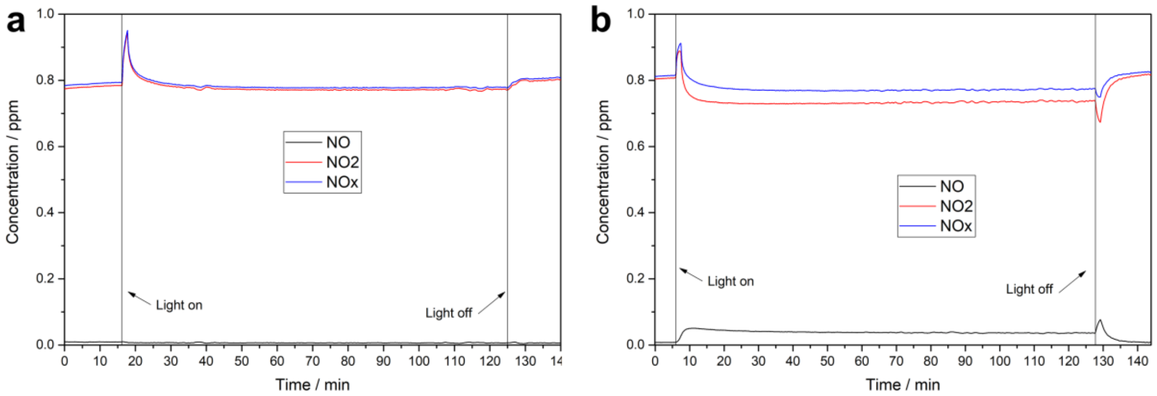

Figure S25. Photocatalytic conversion of gaseous NO_2. Concentration of NO, NO_2 and total NO_x during photocatalytic conversion of NO_2 over a-TiO_2(400) (a) and a-TiO_2(400)-V^{SA} (b). Gas flow rate 3 L/min, irradiance 1 mW cm^{-2} (365 nm LED).

3. Computational details

All of the theoretical calculations were carried out using VASP quantum mechanical tool[14] under spin-polarized density functional theory[15] on TiO_2(101). The calculations were performed using the Generalized Gradient Approach (GGA) of Perdew, Burke, and Ernzerhof (PBE),[16] PBE corrected for on-site Coulomb interactions (PBE + U).[17] We have used the PBE+U method.[18] U values represent exact exchange in an approximation to correct for the large self-interaction error incurred by PBE. The effective on-site Coulomb U parameters are 4eV for d-electrons for Ti atom,[19] 2eV for V atom, [20] and 5eV for Cu atom.[21] We used PBE+U since this method is very well-known for its good performance in modeling catalysis and photocatalysis in general, and TiO_2 in particular.[19] We used a k-mesh of 1×1×1 gamma point since the supercell size is large enough and an energy cutoff of 600 eV for the plane-wave basis set. These k-grid's and energy cutoffs were converged to the total energy within 1 meV/atom. For all the calculations, we relaxed the cells using a convergence criterion of 10^{-6} eV for electronic iterations and 0.03 eV/Å for ionic iterations. Geometrical relaxations took place with a conjugate gradient algorithm. All slabs were separated from their periodic image by a minimum of 14 Å vacuum layers which is converged the total energy up to 0.1 meV/atom.

<u>Cell Structures</u>:

TiO_2-anatase(101) [Energy(DFT+U) = -1777.6955eV]

```
   1.00000000000000
    21.8327007293999991    0.0000000000000000    0.0000000000000000
    -6.5600887955999996   17.7489792478999995    0.0000000000000000
     0.0000000000000000    0.0000000000000000   23.8589000702000007
   Ti   O
    80   160
Direct
  0.1298693262341217  0.1915009507791225  0.0308565832475907
  0.1990576149371677  0.0191652219504093  0.1861733900230504
  0.0151123204014212  0.0455938875245394  0.0593966004330397
  0.0843088461027364  0.0732718224172686  0.2147324805352753
  0.3798693112341240  0.1915009507791225  0.0308565832475907
  0.4490576149371677  0.0191652219504093  0.1861733900230504
  0.2651123164014209  0.0455938875245394  0.0593966004330397
  0.3343088531027334  0.0732718224172686  0.2147324805352753
  0.6298693412341194  0.1915009507791225  0.0308565832475907
  0.6990576449371702  0.0191652219504093  0.1861733900230504
  0.5151123464014233  0.0455938875245394  0.0593966004330397
  0.5843088231027380  0.0732718224172686  0.2147324805352753
  0.8798693412341194  0.1915009507791225  0.0308565832475907
  0.9490576449371702  0.0191652219504093  0.1861733900230504
  0.7651123464014233  0.0455938875245394  0.0593966004330397
  0.8343088231027380  0.0732718224172686  0.2147324805352753
  0.1298693262341217  0.3915009387791244  0.0308565832475907
  0.1990576149371677  0.2191652209504156  0.1861733900230504
```

0.0151123204014212	0.2455938935245356	0.0593966004330397
0.0843088461027364	0.2732718104172633	0.2147324805352753
0.3798693112341240	0.3915009387791244	0.0308565832475907
0.4490576149371677	0.2191652209504156	0.1861733900230504
0.2651123164014209	0.2455938935245356	0.0593966004330397
0.3343088531027334	0.2732718104172633	0.2147324805352753
0.6298693412341194	0.3915009387791244	0.0308565832475907
0.6990575849371652	0.2191652209504156	0.1861733900230504
0.5151123464014233	0.2455938935245356	0.0593966004330397
0.5843088231027380	0.2732718104172633	0.2147324805352753
0.8798693412341194	0.3915009387791244	0.0308565832475907
0.9490575849371652	0.2191652209504156	0.1861733900230504
0.7651123464014233	0.2455938935245356	0.0593966004330397
0.8343088231027380	0.2732718104172633	0.2147324805352753
0.1298693262341217	0.5915009867791241	0.0308565832475907
0.1990576149371677	0.4191652389504128	0.1861733900230504
0.0151123264014217	0.4455938965245352	0.0593966004330397
0.0843088531027334	0.4732718284172677	0.2147324805352753
0.3798693412341194	0.5915009867791241	0.0308565832475907
0.4490576149371677	0.4191652389504128	0.1861733900230504
0.2651123164014209	0.4455938965245352	0.0593966004330397
0.3343088531027334	0.4732718284172677	0.2147324805352753
0.6298693412341194	0.5915009867791241	0.0308565832475907
0.6990576449371702	0.4191652389504128	0.1861733900230504
0.5151123464014233	0.4455938965245352	0.0593966004330397
0.5843088831027359	0.4732718284172677	0.2147324805352753
0.8798693412341194	0.5915009867791241	0.0308565832475907
0.9490575849371652	0.4191652389504128	0.1861733900230504
0.7651123464014233	0.4455938965245352	0.0593966004330397
0.8343088831027359	0.4732718284172677	0.2147324805352753
0.1298693112341240	0.7915009157791246	0.0308565832475907
0.1990576149371677	0.6191651669504097	0.1861733900230504
0.0151123224014142	0.6455939145245395	0.0593966004330397
0.0843088461027364	0.6732718164172695	0.2147324805352753
0.3798693112341240	0.7915009157791246	0.0308565832475907
0.4490576149371677	0.6191651669504097	0.1861733900230504
0.2651123164014209	0.6455939145245395	0.0593966004330397
0.3343088531027334	0.6732718164172695	0.2147324805352753
0.6298692822341252	0.7915009157791246	0.0308565832475907
0.6990576449371702	0.6191651669504097	0.1861733900230504
0.5151123464014233	0.6455939145245395	0.0593966004330397
0.5843088231027380	0.6732718164172695	0.2147324805352753
0.8798693412341194	0.7915009157791246	0.0308565832475907
0.9490575849371652	0.6191651669504097	0.1861733900230504
0.7651123464014233	0.6455939145245395	0.0593966004330397
0.8343088831027359	0.6732718164172695	0.2147324805352753
0.1298693412341194	0.9915010227791257	0.0308565832475907
0.1990576149371677	0.8191652149504094	0.1861733900230504
0.0151123244014215	0.8455939025245343	0.0593966004330397
0.0843088531027334	0.8732718044172643	0.2147324805352753
0.3798693412341194	0.9915010227791257	0.0308565832475907
0.4490576149371677	0.8191652149504094	0.1861733900230504
0.2651123164014209	0.8455939025245343	0.0593966004330397
0.3343088531027334	0.8732718044172643	0.2147324805352753

0.6298693412341194	0.9915010227791257	0.0308565832475907
0.6990576449371702	0.8191652149504094	0.1861733900230504
0.5151123464014233	0.8455939025245343	0.0593966004330397
0.5843088231027380	0.8732718044172643	0.2147324805352753
0.8798693412341194	0.9915010227791257	0.0308565832475907
0.9490576449371702	0.8191652149504094	0.1861733900230504
0.7651122874014220	0.8455939025245343	0.0593966004330397
0.8343088231027380	0.8732718044172643	0.2147324805352753
0.0366441903421659	0.1542071408130425	0.0607439104465826
0.1099106382289960	0.1835135333649944	0.2262737115389655
0.1768464924249429	0.0102895219088666	0.0983706030582780
0.2530568283296262	0.0407752227039282	0.2505777506463716
0.1042846418351218	0.0812676087598661	0.0192837249107569
0.1775184756467425	0.1105564579212057	0.1847756328611823
0.2111383513293745	0.0240085577655549	0.9950015830620700
0.0373222651875977	0.0544700770912172	0.1472140262368455
0.2866441833421689	0.1542071408130425	0.0607439104465826
0.3599106382289960	0.1835135333649944	0.2262737115389655
0.4268464924249429	0.0102895219088666	0.0983706030582780
0.5030568133296285	0.0407752227039282	0.2505777506463716
0.3542846488351188	0.0812676087598661	0.0192837249107569
0.4275184756467425	0.1105564579212057	0.1847756328611823
0.4611383663293722	0.0240085577655549	0.9950015830620700
0.2873222581876007	0.0544700770912172	0.1472140262368455
0.5366442433421668	0.1542071408130425	0.0607439104465826
0.6099106382289960	0.1835135333649944	0.2262737115389655
0.6768464924249429	0.0102895219088666	0.0983706030582780
0.7530567833296260	0.0407752227039282	0.2505777506463716
0.6042846188351163	0.0812676087598661	0.0192837249107569
0.6775184756467425	0.1105564579212057	0.1847756328611823
0.7111383363293697	0.0240085577655549	0.9950015830620700
0.5373222581876007	0.0544700770912172	0.1472140262368455
0.7866441833421689	0.1542071408130425	0.0607439104465826
0.8599105782289982	0.1835135333649944	0.2262737115389655
0.9268464324249450	0.0102895219088666	0.0983706030582780
0.0030568433296239	0.0407752227039282	0.2505777506463716
0.8542846188351163	0.0812676087598661	0.0192837249107569
0.9275184756467425	0.1105564579212057	0.1847756328611823
0.9611383963293747	0.0240085577655549	0.9950015830620700
0.7873223171875949	0.0544700770912172	0.1472140262368455
0.0366441943421663	0.3542071588130398	0.0607439104465826
0.1099106382289960	0.3835135063649915	0.2262737115389655
0.1768464924249429	0.2102895339088704	0.0983706030582780
0.2530568433296239	0.2407752327039248	0.2505777506463716
0.1042846338351211	0.2812676047598686	0.0192837249107569
0.1775184756467425	0.3105564829212000	0.1847756328611823
0.2111383663293722	0.2240085657655584	0.9950015830620700
0.0373222651875977	0.2544700840912171	0.1472140262368455
0.2866441833421689	0.3542071588130398	0.0607439104465826
0.3599106382289960	0.3835135063649915	0.2262737115389655
0.4268464924249429	0.2102895339088704	0.0983706030582780
0.5030568433296239	0.2407752327039248	0.2505777506463716
0.3542846488351188	0.2812676047598686	0.0192837249107569
0.4275184756467425	0.3105564829212000	0.1847756328611823

0.4611383663293722	0.2240085657655584	0.9950015830620700
0.2873222581876007	0.2544700840912171	0.1472140262368455
0.5366441833421689	0.3542071588130398	0.0607439104465826
0.6099106382289960	0.3835135063649915	0.2262737115389655
0.6768464924249429	0.2102895339088704	0.0983706030582780
0.7530568433296239	0.2407752327039248	0.2505777506463716
0.6042846788351213	0.2812676047598686	0.0192837249107569
0.6775184756467425	0.3105564829212000	0.1847756328611823
0.7111383363293697	0.2240085657655584	0.9950015830620700
0.5373222581876007	0.2544700840912171	0.1472140262368455
0.7866442433421668	0.3542071588130398	0.0607439104465826
0.8599105782289982	0.3835135063649915	0.2262737115389655
0.9268464924249429	0.2102895339088704	0.0983706030582780
0.0030568433296239	0.2407752327039248	0.2505777506463716
0.8542846188351163	0.2812676047598686	0.0192837249107569
0.9275184756467425	0.3105564829212000	0.1847756328611823
0.9611383963293747	0.2240085657655584	0.9950015830620700
0.7873222581876007	0.2544700840912171	0.1472140262368455
0.0366441903421659	0.5542071468130416	0.0607439104465826
0.1099106452289931	0.5835135543649912	0.2262737115389655
0.1768464924249429	0.4102895229088688	0.0983706030582780
0.2530568283296262	0.4407752207039266	0.2505777506463716
0.1042846418351218	0.4812676227598658	0.0192837249107569
0.1775184606467377	0.5105564419212030	0.1847756328611823
0.2111383513293745	0.4240085537655602	0.9950015830620700
0.0373222731875984	0.4544701010912178	0.1472140262368455
0.2866441833421689	0.5542071468130416	0.0607439104465826
0.3599106382289960	0.5835135543649912	0.2262737115389655
0.4268465224249454	0.4102895229088688	0.0983706030582780
0.5030568433296239	0.4407752207039266	0.2505777506463716
0.3542846488351188	0.4812676227598658	0.0192837249107569
0.4275184756467425	0.5105564419212030	0.1847756328611823
0.4611383663293722	0.4240085537655602	0.9950015830620700
0.2873222581876007	0.4544701010912178	0.1472140262368455
0.5366441833421689	0.5542071468130416	0.0607439104465826
0.6099106382289960	0.5835135543649912	0.2262737115389655
0.6768464924249429	0.4102895229088688	0.0983706030582780
0.7530568433296239	0.4407752207039266	0.2505777506463716
0.6042846188351163	0.4812676227598658	0.0192837249107569
0.6775184756467425	0.5105564419212030	0.1847756328611823
0.7111383363293697	0.4240085537655602	0.9950015830620700
0.5373223171875949	0.4544701010912178	0.1472140262368455
0.7866441833421689	0.5542071468130416	0.0607439104465826
0.8599106382289960	0.5835135543649912	0.2262737115389655
0.9268464924249429	0.4102895229088688	0.0983706030582780
0.0030568433296239	0.4407752207039266	0.2505777506463716
0.8542846788351213	0.4812676227598658	0.0192837249107569
0.9275184756467425	0.5105564419212030	0.1847756328611823
0.9611383963293747	0.4240085537655602	0.9950015830620700
0.7873222581876007	0.4544701010912178	0.1472140262368455
0.0366441983421666	0.7542071938130377	0.0607439104465826
0.1099106382289960	0.7835134823649952	0.2262737115389655
0.1768464924249429	0.6102895109088706	0.0983706030582780
0.2530568433296239	0.6407752387039238	0.2505777506463716

0.1042846338351211	0.6812676107598676	0.0192837249107569
0.1775184756467425	0.7105564299212048	0.1847756328611823
0.2111383363293697	0.6240085117655596	0.9950015830620700
0.0373222651875977	0.6544700600912208	0.1472140262368455
0.2866442133421643	0.7542071938130377	0.0607439104465826
0.3599106382289960	0.7835134823649952	0.2262737115389655
0.4268464924249429	0.6102895109088706	0.0983706030582780
0.5030568433296239	0.6407752387039238	0.2505777506463716
0.3542846488351188	0.6812676107598676	0.0192837249107569
0.4275184756467425	0.7105564299212048	0.1847756328611823
0.4611383363293697	0.6240085117655596	0.9950015830620700
0.2873222581876007	0.6544700600912208	0.1472140262368455
0.5366441833421689	0.7542071938130377	0.0607439104465826
0.6099106382289960	0.7835134823649952	0.2262737115389655
0.6768464924249429	0.6102895109088706	0.0983706030582780
0.7530568433296239	0.6407752387039238	0.2505777506463716
0.6042846188351163	0.6812676107598676	0.0192837249107569
0.6775184756467425	0.7105564299212048	0.1847756328611823
0.7111383363293697	0.6240085117655596	0.9950015830620700
0.5373222581876007	0.6544700600912208	0.1472140262368455
0.7866441833421689	0.7542071938130377	0.0607439104465826
0.8599106382289960	0.7835134823649952	0.2262737115389655
0.9268464924249429	0.6102895109088706	0.0983706030582780
0.0030569023296252	0.6407752387039238	0.2505777506463716
0.8542846188351163	0.6812676107598676	0.0192837249107569
0.9275184756467425	0.7105564299212048	0.1847756328611823
0.9611383363293697	0.6240085117655596	0.9950015830620700
0.7873222581876007	0.6544700600912208	0.1472140262368455
0.0366441983421666	0.9542071228130382	0.0607439104465826
0.1099106302289954	0.9835134713649936	0.2262737115389655
0.1768464924249429	0.8102894989088654	0.0983706030582780
0.2530568283296262	0.8407752267039257	0.2505777506463716
0.1042846418351218	0.8812675987598695	0.0192837249107569
0.1775184756467425	0.9105564769212009	0.1847756328611823
0.2111383513293745	0.8240085597655593	0.9950015830620700
0.0373222731875984	0.8544701070912168	0.1472140262368455
0.2866441833421689	0.9542071228130382	0.0607439104465826
0.3599106382289960	0.9835134713649936	0.2262737115389655
0.4268464624249404	0.8102894989088654	0.0983706030582780
0.5030568433296239	0.8407752267039257	0.2505777506463716
0.3542846488351188	0.8812675987598695	0.0192837249107569
0.4275184756467425	0.9105564769212009	0.1847756328611823
0.4611383663293722	0.8240085597655593	0.9950015830620700
0.2873222581876007	0.8544701070912168	0.1472140262368455
0.5366441833421689	0.9542071228130382	0.0607439104465826
0.6099106382289960	0.9835134713649936	0.2262737115389655
0.6768464924249429	0.8102894989088654	0.0983706030582780
0.7530568433296239	0.8407752267039257	0.2505777506463716
0.6042846788351213	0.8812675987598695	0.0192837249107569
0.6775184756467425	0.9105564769212009	0.1847756328611823
0.7111383363293697	0.8240085597655593	0.9950015830620700
0.5373222581876007	0.8544701070912168	0.1472140262368455
0.7866441833421689	0.9542071228130382	0.0607439104465826
0.8599106382289960	0.9835134713649936	0.2262737115389655

0.9268464924249429 0.8102894989088654 0.0983706030582780
0.0030567833296260 0.8407752267039257 0.2505777506463716
0.8542846188351163 0.8812675987598695 0.0192837249107569
0.9275184756467425 0.9105564769212009 0.1847756328611823
0.9611383363293697 0.8240085597655593 0.9950015830620700
0.7873222581876007 0.8544701070912168 0.1472140262368455

CuO at TiO_2-anatase(101) [Energy(DFT+U) = -1784.3790eV]
1.0000000000000
21.8327007293999991 0.0000000000000000 0.0000000000000000
-6.5600887955999996 17.7489792478999995 0.0000000000000000
0.0000000000000000 0.0000000000000000 23.8589000702000007
Ti O Cu
80 161 1
Direct
0.1284323230882691 0.1912908703899774 0.0307811429306000
0.1996662830838076 0.0192903805708795 0.1856145025711626
0.0140918057733614 0.0453659481100672 0.0590883747233590
0.0851661703840705 0.0735773201025367 0.2139032917789478
0.3784834260234931 0.1912519453277923 0.0308339019564272
0.4502981663847478 0.0195445034501560 0.1857033246684168
0.2639103809200591 0.0449906079160058 0.0584963858807726
0.3351037229096931 0.0728832100152914 0.2133326578560926
0.6289421380061953 0.1914658234422006 0.0317457818576159
0.7002520556225562 0.0197343454492298 0.1864086845225827
0.5141754530238458 0.0453703050889871 0.0588427360744745
0.5854587232306869 0.0737939019958205 0.2138275959513507
0.8789608656280308 0.1912631586417959 0.0311934501379056
0.9498697239080514 0.0193417393436306 0.1861508181902352
0.7642938644823474 0.0453761471389171 0.0593704200437983
0.8355786102994429 0.0738695619181229 0.2140955584840114
0.1315226236038711 0.3921610387634971 0.0320594984892821
0.1996935217405991 0.2191486587942109 0.1858168472075903
0.0144437050057178 0.2459952782342896 0.0587233111905050
0.0861132606634385 0.2748144920816102 0.2134435778082135
0.3769071938865451 0.3902783434224872 0.0302954970130926
0.4502799546035590 0.2187724588743478 0.1860331871410565
0.2634956753377935 0.2447473916572633 0.0589775208561392
0.3348112544809041 0.2711259199648524 0.2134031294053784
0.6288286720651044 0.3910742855867042 0.0313449292914711
0.7002958161058643 0.2198616566620188 0.1864923588455483
0.5137933935603129 0.2451960713055215 0.0589737234039021
0.5849875056537854 0.2734448839238866 0.2143215060884813
0.8790466221626190 0.3912590778160308 0.0312866590416974
0.9506035409849574 0.2197505951453422 0.1860048226305508
0.7643496989765453 0.2454256814424340 0.0593551572875484
0.8357001614807160 0.2737822367617468 0.2140667659680062
0.1286785380887920 0.5907094997874580 0.0309733286444001
0.2037079621899878 0.4212368018493038 0.1883498262028169
0.0145022015927765 0.4447131911853361 0.0589503081150724
0.0858499811618216 0.4731907500233206 0.2137078494240328
0.3769751609704173 0.5902285858128735 0.0304012724385032
0.4446657975360395 0.4180555279236557 0.1814531573680256
0.2650246403935199 0.4455034118736449 0.0686346926026928

0.3371697075721016	0.4740636740352073	0.2352363013528063
0.6286578544125447	0.5909838777963969	0.0311816674468091
0.6994060484504629	0.4196552518615064	0.1867603261586410
0.5136297928991667	0.4449947642736092	0.0572931503320220
0.5839805658370807	0.4738187067830495	0.2125507190003333
0.8787309629350304	0.5910301620049196	0.0312400030023809
0.9503173985151747	0.4199111480039122	0.1861805424217593
0.7640593377900018	0.4451465400612236	0.0595221057674777
0.8349903676535391	0.4735790529347810	0.2145326729206616
0.1285995456923814	0.7909388062111375	0.0308009339842386
0.1997875990971707	0.6196344139114345	0.1859655460436116
0.0141427346901466	0.6449695809636395	0.0590604666797745
0.0851392434273066	0.6735096779932661	0.2139420831697265
0.3785260004308668	0.7907594359230004	0.0306593543876943
0.4452721488589049	0.6171026139508626	0.1817368841508440
0.2637468860563601	0.6452161385889923	0.0592136115712378
0.3349569371753134	0.6752581412289729	0.2139144435803786
0.6287306836560163	0.7911755748535825	0.0310686874524535
0.7001234047458169	0.6196127930553601	0.1858272657661644
0.5136809405006559	0.6450879085842516	0.0572470884768279
0.5847129483578470	0.6733025753311352	0.2120432123009337
0.8787520412660399	0.7909961701296169	0.0314199784775298
0.9498305951271178	0.6194170876534173	0.1861147767964937
0.7641036031508079	0.6451026390059980	0.0589290528091126
0.8352511035159509	0.6735896976058484	0.2142968897193001
0.1285433697753078	0.9909956171306504	0.0307010206772489
0.1996977905465158	0.8194376383326372	0.1856688900380092
0.0141042551084709	0.8451768566622775	0.0593227790271200
0.0851530828296134	0.8736027335429029	0.2142600082159802
0.3790362880113136	0.9912107794235467	0.0307738102412358
0.4502286664572779	0.8202353852460789	0.1857202777838864
0.2639857938039469	0.8453157193835850	0.0585636094233948
0.3351470082865475	0.8739682080464490	0.2135244250985480
0.6289979688512588	0.9912584130888291	0.0315691307789479
0.6996594230843129	0.8194590525133094	0.1862414950047011
0.5138246139883478	0.8452271169153462	0.0585864246972108
0.5850491148793395	0.8738311021105147	0.2139175558677380
0.8787612947418708	0.9910379509888756	0.0312168057705691
0.9498483730264127	0.8194731743599135	0.1863800803659856
0.7639884360818812	0.8451301242322558	0.0591461194236729
0.8349957857374477	0.8735231102155510	0.2143690756841536
0.0351094034010373	0.1537212380857866	0.0602746069918609
0.1106239108110998	0.1833742791676727	0.2251630365793034
0.1761877772737037	0.0099232990185527	0.0978410074013709
0.2542336655032216	0.0408522919935024	0.2496935463151289
0.1030062928155715	0.0807129024980924	0.0191801226244408
0.1778463345660413	0.1101289649262327	0.1836346235670021
0.2095335200026014	0.0228782795513141	0.9943157342687883
0.0374924486744206	0.0543585361304508	0.1466443333754768
0.2850336690615407	0.1533662028798872	0.0593516804316252
0.3618181799302889	0.1826544887076054	0.2257729366829366
0.4264287497351305	0.0099826819594142	0.0979967484997957
0.5045053778285364	0.0414353025634782	0.2500147792181338
0.3536008378811601	0.0811381918725829	0.0193752666791553

0.4285491786500870	0.1104354042518310	0.1830341053802158
0.4601066102798157	0.0238294997563457	0.9945167806654283
0.2876563845307203	0.0545992951009495	0.1461084024327945
0.5351728162572797	0.1539056518644415	0.0607023954080574
0.6109842829930088	0.1839751053321663	0.2256543697622391
0.6762574200409972	0.0097456547952746	0.0988687514150897
0.7544332841049410	0.0415635200140656	0.2504759870957542
0.6036687621698817	0.0812469345073339	0.0200924955916406
0.6790004009828650	0.1112904408557682	0.1847057743426319
0.7098190051767261	0.0235846557859958	0.9951910926763006
0.5382848675372074	0.0547956337701692	0.1464447022317756
0.7852470760778090	0.1537326680241264	0.0609525706990155
0.8609330809584748	0.1838192877120761	0.2256723276954489
0.9260361984321719	0.0098105237327530	0.0984273864814540
0.0039578261495095	0.0407294561928282	0.2502452175109013
0.8531365498154742	0.0807697497068176	0.0197617229603679
0.9284685216256960	0.1107337701729776	0.1844407702362290
0.9595532685394517	0.0231915454512119	0.9948772344516001
0.7879016306246100	0.0545239067921912	0.1468887545004378
0.0355599286111357	0.3538251722683086	0.0600474649934881
0.1096691624935318	0.3835126790555421	0.2256577290587956
0.1758827519026411	0.2102311034329389	0.0981228697118013
0.2543842960768572	0.2391060761361032	0.2500113305751910
0.1036015442841176	0.2811338375578103	0.0194447359237415
0.1796515584564773	0.3109184007271466	0.1860634803076024
0.2095130374561407	0.2239861706917310	0.9946107016885861
0.0380547937958085	0.2550600270775263	0.1462607107701146
0.2862477012001534	0.3532481826703062	0.0631867990370054
0.3584920009780959	0.3796511648117828	0.2201999459142101
0.4259963266133866	0.2115524750592925	0.0983358318589467
0.5043336694340397	0.2390213593886656	0.2505994042575850
0.3523511068472800	0.2806003374283392	0.0178634559690565
0.4315067738474596	0.3118621422471719	0.1869498762933333
0.4597318513495523	0.2233120759426868	0.9947726485402129
0.2875969461252694	0.2508817422140268	0.1461483927361300
0.5350575937464797	0.3539075048183733	0.0598020875976530
0.6081582889052370	0.3842447336308297	0.2261037514569679
0.6763614040095121	0.2109987822047898	0.0989773126233828
0.7545450078631362	0.2413491695062504	0.2505061264204258
0.6031792572900017	0.2810287816718784	0.0196766962455470
0.6785005161383566	0.3117282503404013	0.1860317801870366
0.7097211532919232	0.2237343285020259	0.9952910905663046
0.5381238674379176	0.2547767028695773	0.1467795495953084
0.7854772900072220	0.3539849662131758	0.0611835046001517
0.8604780818620839	0.3839651648308688	0.2260155677918974
0.9263813468726951	0.2104405859606544	0.0982911479816195
0.0046294886038325	0.2408708894037943	0.2498506846836932
0.8529950944510034	0.2808682690925082	0.0195782732111383
0.9286307135369825	0.3111360345201390	0.1847485405000810
0.9595145997211745	0.2235182214273834	0.9945950007178084
0.7879083987755280	0.2549257550577479	0.1469423394914955
0.0354232921992050	0.5536199397724602	0.0604457237903944
0.1106511101692362	0.5842191169649738	0.2254282953732059
0.1747055549647811	0.4092222272258255	0.1015524728195132

0.2493921081045940	0.4399745378402997	0.2567555573507789
0.1038802377700563	0.4809969960748290	0.0197884979243810
0.1790960931003056	0.5118456174508168	0.1857671840148498
0.2129538324654092	0.4246770775298074	0.9998944734288031
0.0377755534583528	0.4545369398005050	0.1465046678429829
0.2861026109247149	0.5546976712337255	0.0628336395253015
0.3601831769868795	0.5868076269956575	0.2231522137150606
0.4256208927554113	0.4075025982298897	0.0975201148848228
0.5020648208821754	0.4548383243282501	0.2510255008601519
0.3530607417462832	0.4806735742970929	0.0227733004391553
0.4254567356405232	0.5099644422703520	0.1828662601836868
0.4586056733903732	0.4243031559877011	0.9941354579777553
0.2872559088859319	0.4549367186392814	0.1516490646733288
0.5352387377683954	0.5536338309070032	0.0615335003473092
0.6107827687327827	0.5841434888118826	0.2251882524279196
0.6757498809554576	0.4097746540338747	0.0988062467106232
0.7534875077510748	0.4421867867573965	0.2507630889983403
0.6028038199125518	0.4809112969044875	0.0188187329899705
0.6762743615003686	0.5104390472015865	0.1838897944069515
0.7097294111664922	0.4237402290371293	0.9952689022068810
0.5358198732498849	0.4521404712759747	0.1465255191905541
0.7852906080103921	0.5537313965315462	0.0612127529266999
0.8604802720697933	0.5838770026570188	0.2262750689859416
0.9263119252247449	0.4098698206178213	0.0984171021760432
0.0043005907672296	0.4421799557807162	0.2500326829142594
0.8529942862472382	0.4809084030356630	0.0197996533665474
0.9275645081818027	0.5108238656893462	0.1843049238488348
0.9596366386346986	0.4235380355074909	0.9948087763202906
0.7868246643106005	0.4546740471927464	0.1474650981880288
0.0354907091384860	0.7536568902870897	0.0607708399388898
0.1107158979943179	0.7839678010546436	0.2258483831271789
0.1760906473465838	0.6099864381739124	0.0983258012104784
0.2543530420740296	0.6431534972096813	0.2502570237902333
0.1030691342946639	0.6808009778931847	0.0191720618311351
0.1779663869585448	0.7111689535945231	0.1837140016676528
0.2097083537499387	0.6229046264166413	0.9948217537026451
0.0373947410777120	0.6544009532016872	0.1466858636125963
0.2853923349723431	0.7539082332929254	0.0597204781430989
0.3618818916275899	0.7854275105159942	0.2257958187785576
0.4257490913528201	0.6117956627263794	0.0975408598739023
0.5033452703489019	0.6279706202327446	0.2508998895544963
0.3525732880004284	0.6804533780189175	0.0180828317016406
0.4309458974445874	0.7120959165398872	0.1858709385814237
0.4585945647662015	0.6215910861271254	0.9941163839708622
0.2878037451954327	0.6571657461733764	0.1465098187823628
0.5349118194008753	0.7533710011504056	0.0593391867759152
0.6087422964581606	0.7820277394384405	0.2256129092530870
0.6760205631943990	0.6100030208202725	0.0984362949648414
0.7536435286870642	0.6409975231453657	0.2502471054387030
0.6028341127325518	0.6804450748825133	0.0187633018871978
0.6770361226785866	0.7102156571943183	0.1836438348682421
0.7094559339792283	0.6231021472615552	0.9948442601127994
0.5361262731940712	0.6558456347449138	0.1462262488698158
0.7851792917010982	0.7535065425703351	0.0609655112242535

0.8604450608985772 0.7836758947122817 0.2262471905801675
0.9260197582535739 0.6102030051162615 0.0984678009758042
0.0039728654562126 0.6411066247960449 0.2502586677358423
0.8528594178415005 0.6807106514251871 0.0193278149126144
0.9280447822178743 0.7107792957913190 0.1845965929181901
0.9594856894518458 0.6232780806306479 0.9948941544623224
0.7875806477678253 0.6544740443662889 0.1469314416464087
0.0354219505614637 0.9537036244282433 0.0607488711878901
0.1108187066527151 0.9836591672697423 0.2259429301684648
0.1762564141126504 0.8101262318916369 0.0979510843077733
0.2542212337933307 0.8412933356521961 0.2498325845531681
0.1027844364718220 0.8806315493365560 0.0192138110181617
0.1780500668345795 0.9106939867468640 0.1838202712652688
0.2096706194853795 0.8238137700392940 0.9944548763221519
0.0375258488368075 0.8546316074678018 0.1469738409093964
0.2857709689739139 0.9538808600370032 0.0601382164905075
0.3613802884459432 0.9839660192482782 0.2257944716765294
0.4261318718144693 0.8088494449091215 0.0980548683151383
0.5044129653687506 0.8425905550744517 0.2502389732217054
0.3534200709427822 0.8808877563142872 0.0192185170134707
0.4285897388764752 0.9112363356838102 0.1831979643636927
0.4596971690454978 0.8237353819730870 0.9944501564680692
0.2877101073394996 0.8546426493549220 0.1462729823547804
0.5352987298107621 0.9536867983707964 0.0602575531790563
0.6110124235087895 0.9839742531926632 0.2256445745224838
0.6757990667551397 0.8099049090763017 0.0984874276997658
0.7534701077484129 0.8400733710788302 0.2504558643156543
0.6033401796637250 0.8809258280516374 0.0196712016918212
0.6785770110066451 0.9105248187510142 0.1854075953960148
0.7096759242665129 0.8231033134030810 0.9949013990903737
0.5381924192714536 0.8551348722204608 0.1464378281414724
0.7852584561194860 0.9535158509329662 0.0608614046118632
0.8604879490905191 0.9835597473136346 0.2259779445770675
0.9260383361161004 0.8097791441681110 0.0986923639837514
0.0039688234399549 0.8410545070757038 0.2505328868691237
0.8528859671913125 0.8805732037620828 0.0197291975987639
0.9277867217879745 0.9104700758687727 0.1844789376224654
0.9595877349655666 0.8233400662475177 0.9951361337584785
0.7872120964987062 0.8542863660159412 0.1471179199658366
0.3823516061458108 0.4875557821872007 0.3026525709137715
0.4670759094190515 0.5272098291030716 0.2823824492852651

VO_2 at TiO_2-anatase(101) [Energy(DFT+U) = -1806.6885 eV]
1.00000000000000
21.8327007293999991 0.0000000000000000 0.0000000000000000
-6.5600887955999996 17.7489792478999995 0.0000000000000000
0.0000000000000000 0.0000000000000000 23.8589000702000007
Ti O V H
80 162 1 2
Direct

0.1301561222906642	0.1920244195508687	0.0317704945927701
0.2005905930163152	0.0196510135198338	0.1859883187313329
0.0151116007721370	0.0457085928586025	0.0584714225121630
0.0857780044160776	0.0735541690974415	0.2137139803719421
0.3797654669215973	0.1919504818184947	0.0320680228484420
0.4514285631322537	0.0204173929974232	0.1863276780398238
0.2649512019556468	0.0457665930925302	0.0591490155544534
0.3359741005986194	0.0736988452930234	0.2141971159125120
0.6297364801341416	0.1913612970964280	0.0313986593828162
0.7014905305786385	0.0199946405020199	0.1857515990683467
0.5150737637986467	0.0455412860360269	0.0593108895909964
0.5867755104107815	0.0738393244349282	0.2143325894952710
0.8801404299023972	0.1915100659989122	0.0308879867834619
0.9507549265613662	0.0195025787175780	0.1853314086565092
0.7652017448819208	0.0454633828841438	0.0586086401670869
0.8366768472607617	0.0740848510075693	0.2136892907300023
0.1315177635676079	0.3923240980177241	0.0341220936425159
0.2006941411980989	0.2192184055838879	0.1870533133714645
0.0154246049213000	0.2460548668639859	0.0591413627768915
0.0866237338178308	0.2743136475758163	0.2142982056807554
0.3794914878294122	0.3922248201043104	0.0321415205548092
0.4518446672810725	0.2192122080183552	0.1873924806013676
0.2651801368607423	0.2459587632265539	0.0604813811076070
0.3357475259927512	0.2720009939361034	0.2145115687542969
0.6291913148036059	0.3912591452394381	0.0325680062726761
0.7019306798923282	0.2195919140918505	0.1859888814594939
0.5149687050315421	0.2453765735439859	0.0602968101833170
0.5874641617383887	0.2726461356968102	0.2149216042548403
0.8798502645120365	0.3914392523687269	0.0314361574530437
0.9515603695897425	0.2197737981352788	0.1858952085286703
0.7651726549144513	0.2453491977884070	0.0588643965648927
0.8371510015723373	0.2740037395053392	0.2137804488824102
0.1297179320173214	0.5910265877912053	0.0321372075289688
0.2029873770342903	0.4206213282330182	0.1891122515532331
0.0151511807174387	0.4452500138887814	0.0595504208076179
0.0866429351554530	0.4738591996260908	0.2146142786810401
0.3782205149394073	0.5903647363592199	0.0323993401680909
0.4497349087293472	0.4180852366178911	0.1849620881937426
0.2660021684377725	0.4458678082339986	0.0667763147428602
0.3361443513271780	0.4737893386999659	0.2271835869235304
0.6292437960263015	0.5907222936981142	0.0319926534289365
0.7016637750008741	0.4191483741993380	0.1870322150338595
0.5149227036050874	0.4456289885722455	0.0640087581938076
0.5887608975980356	0.4720832118691547	0.2200579155899973
0.8794500650595012	0.5910038479613959	0.0311521463479707
0.9515791612821474	0.4200115277354470	0.1862692890436151
0.7647685084430123	0.4452304217112584	0.0597428672764337
0.8368653650091034	0.4736825812923016	0.2142852944889242
0.1295186429817434	0.7912568114090774	0.0309995682082018
0.2004088479805901	0.6203063628841718	0.1872636432063999
0.0148702978313793	0.6453804055030119	0.0589956628457387
0.0855725213708780	0.6741826278379364	0.2140878767566790
0.3792962663141921	0.7911631768702208	0.0317402237906208
0.4475275695119620	0.6202851243390626	0.1833942842466101

0.2647351220780223	0.6455774481708616	0.0607077277399952
0.3355089998958718	0.6766639906898249	0.2146722894052360
0.6293365681538674	0.7913521749888517	0.0308256123977699
0.7003637208191691	0.6184299874215000	0.1862756885302446
0.5152033146299217	0.6455076550565906	0.0590361872759502
0.5875877894879693	0.6755696294518501	0.2126487128633343
0.8793698645578587	0.7909593666551515	0.0304584832421355
0.9504128666609191	0.6198381465481333	0.1859321564408418
0.7648336673375766	0.6451764318383724	0.0593955755578222
0.8357135464401679	0.6740101389857571	0.2141981330850626
0.1296990308009214	0.9914334060968031	0.0309443907547404
0.2004076702457880	0.8198691843039612	0.1860118607937693
0.0147632102989661	0.8454443399495943	0.0584351798231353
0.0856159947659876	0.8738891090824765	0.2136074535827959
0.3798028962341817	0.9917156598750623	0.0315363040016692
0.4513856750442145	0.8219076629257884	0.1866392591346795
0.2647973761449975	0.8457630370225004	0.0591525988772261
0.3358381925929947	0.8748704098170776	0.2141357858071800
0.6296513268320751	0.9913155899193598	0.0311403857656316
0.7011139141570055	0.8202951229472291	0.1855876524033491
0.5147631672069366	0.8455264952568200	0.0593078684606070
0.5865516366288759	0.8746739394891634	0.2142933336750161
0.8797810593284652	0.9912464795268079	0.0304123315530305
0.9504452936407262	0.8195721653167993	0.1853398924901057
0.7648750972932348	0.8453487926223744	0.0584206118379313
0.8362100369213294	0.8738827747487861	0.2137164039475792
0.0363344269801189	0.1542091380340693	0.0600264179914021
0.1113204985362231	0.1834879675503913	0.2260255160476774
0.1769405795858958	0.0102017164707675	0.0982918570892650
0.2547843657132631	0.0408736649677337	0.2502457304380670
0.1045896297098707	0.0816031969276807	0.0195046398721104
0.1787398373435920	0.1105919429693500	0.1841981516177640
0.2107814955997611	0.0237780671531311	0.9948069267446655
0.0385373734341385	0.0547848821536334	0.1462349910504699
0.2861794823970243	0.1541221708129257	0.0606326097719858
0.3626516871409393	0.1831068635424415	0.2273907283252257
0.4273446572521564	0.0107058320968747	0.0986578716102500
0.5056908143041312	0.0416315994830896	0.2505371268105989
0.3543801047688433	0.0815863141066089	0.0200256808644212
0.4292833127677156	0.1108043469677398	0.1838998764084181
0.4607051221715466	0.0237464567127432	0.9951489505398783
0.2885439999241655	0.0551514689221619	0.1468300132913640
0.5365641803569474	0.1539375649405770	0.0613766952981578
0.6133654290210373	0.1835504783792885	0.2271013077483559
0.6773856190574179	0.0099543138045064	0.0982240706425017
0.7556295509635902	0.0416266613829137	0.2499809982095655
0.6040558697653395	0.0809832402027055	0.0194068918311174
0.6797586858128142	0.1107974528233555	0.1837702587417240
0.7104746572039602	0.0234739518308089	0.9946195583332056
0.5391786423663092	0.0551843208738774	0.1471029996124145
0.7866268720278953	0.1538806715147771	0.0602549955291565
0.8624834422320973	0.1841199541191259	0.2257498969939462
0.9270762775654475	0.0103089032052637	0.0977001702298210
0.0045886195959923	0.0405761619321936	0.2497488358514488

0.8542639665366636	0.0810877088141098	0.0187882239588220
0.9295616177626513	0.1110083860949018	0.1837222729259551
0.9607537531906161	0.0237185779711737	0.9942042850317421
0.7890714353224411	0.0549700815193077	0.1464090845857058
0.0367237480562324	0.3542687389020642	0.0618402862285947
0.1113854380545050	0.3839594338598360	0.2266055496416044
0.1768516427588125	0.2108466511929663	0.0993509830259782
0.2550747715639048	0.2391269320964255	0.2512859591726837
0.1051191943114844	0.2823001001451502	0.0205853992487235
0.1807744876535722	0.3114442838242155	0.1872244649379979
0.2112855404395901	0.2252654703728325	0.9959099313435829
0.0394501589949527	0.2547743168000380	0.1468097895481861
0.2867718901753520	0.3544095791135646	0.0646272035859141
0.3583297764924822	0.3805074469830387	0.2225310246466066
0.4270514075503655	0.2109670325058275	0.0994817263931651
0.5061989906851210	0.2396661162850506	0.2512856641603776
0.3541742921100450	0.2820094474010517	0.0206807915892853
0.4306340378075504	0.3105918093973372	0.1869553356912022
0.4607213781312325	0.2237602481054850	0.9958951805021883
0.2882666280174959	0.2515446296691408	0.1474505578385958
0.5358301964342047	0.3536176902743620	0.0620903497442100
0.6142866198668031	0.3811870592858568	0.2295907078230215
0.6775059931841412	0.2096292439952308	0.0983584377206626
0.7562422564181261	0.2414102207431910	0.2501751596962407
0.6039963646265036	0.2811902700750082	0.0203872546881030
0.6799797546875439	0.3101411792278057	0.1833533276941139
0.7105345471333706	0.2238688920734120	0.9948268218790233
0.5392257699162855	0.2543987566014891	0.1477088100609052
0.7863794265015329	0.3538600393654221	0.0607293103358515
0.8627931737066632	0.3840281939861896	0.2259668633989023
0.9274299510763768	0.2103269386208666	0.0982559663256097
0.0053674204819600	0.2410941945542504	0.2503062194919892
0.8542836720067370	0.2811994936277102	0.0193109032648238
0.9303957026878820	0.3113383897205821	0.1841182110337058
0.9611822642159780	0.2246735572175567	0.9947781349762153
0.7895369992023973	0.2546989771366412	0.1465038258324967
0.0363212164268845	0.5539495701953427	0.0608941893514441
0.1112422412956846	0.5845893962612223	0.2261689825318882
0.1768935839318289	0.4104167707900856	0.1028910213441137
0.2523892964407892	0.4400407220274758	0.2568919978170854
0.1047324508140761	0.4810489388661878	0.0207201553230263
0.1809576669974078	0.5123381129287097	0.1876601626177390
0.2130146380307849	0.4255662707919541	0.0004406269038029
0.0394814381201130	0.4554681946965857	0.1471225478495057
0.2874117680188135	0.5549448888150295	0.0658283555782049
0.3596871303059928	0.5866195195373010	0.2211717566380145
0.4245048880753046	0.4130274435887387	0.1008829867606664
0.4975889418247874	0.4445699110442050	0.2553348180536261
0.3528271503268812	0.4812679729988929	0.0215168004576611
0.4333371118774352	0.5139350296759062	0.1966513596349202
0.4608314252275818	0.4244117271567305	0.9981404022546627
0.2898326554245898	0.4556877081578463	0.1535752612880827
0.5366734808665399	0.5548000799611899	0.0633339974385905
0.6105593848935555	0.5858822881418106	0.2255383096129933

0.6771298424756509	0.4100826202846974	0.0994876445284234
0.7564894767406543	0.4398380130184378	0.2510801972569041
0.6032105203886573	0.4808773464880858	0.0219328718459124
0.6832573918266434	0.5119873086216487	0.1860109904411189
0.7099408882968632	0.4241292041671727	0.9958532875578996
0.5347463150891230	0.4515539240851396	0.1486790145100656
0.7865162657263340	0.5539600242037750	0.0615888748070503
0.8613511437537369	0.5841077485488171	0.2256716582046110
0.9272536391801367	0.4104428481747959	0.0987920769791302
0.0055142576873948	0.4417134758055568	0.2506949900739173
0.8537751924750410	0.4808906814579501	0.0194233301359859
0.9300582133252888	0.5115089532666275	0.1846021408996634
0.9608772271503412	0.4236117958277390	0.9952544269000114
0.7892674204156265	0.4540435791351740	0.1471442475748290
0.0361006860619426	0.7539185436152493	0.0602398406544111
0.1113870272019781	0.7844768567438720	0.2259097195441555
0.1767399513260983	0.6098215186332538	0.0996965300226265
0.2553120739226529	0.6438357944144428	0.2515479263069054
0.1043119463766189	0.6809073613699894	0.0196149546630338
0.1787410551456290	0.7114582977145432	0.1845462309912236
0.2109977133589069	0.6225238493721150	0.9962650396790167
0.0383736736864151	0.6552238380503397	0.1466694354486009
0.2859764650442287	0.7541169271934507	0.0605416229334139
0.3628451444253713	0.7865885113037407	0.2272079928089283
0.4267473434781408	0.6075365415918981	0.1001276711451169
0.5043392749132494	0.6362897874257243	0.2525671637260487
0.3536977774136574	0.6803774428902329	0.0203044642646972
0.4311219817365668	0.7133164035145683	0.1864511441771910
0.4594106807097731	0.6229156681178338	0.9961095657736578
0.2881998142417288	0.6591264271193680	0.1476847833680850
0.5359485428105373	0.7538965676448228	0.0608305784844489
0.6113378940390319	0.7841437653211401	0.2266051568216696
0.6770031105806211	0.6101387179071622	0.0989690770139688
0.7548096225669880	0.6429745680536456	0.2505054697873703
0.6033632454256050	0.6806566666929541	0.0192602082910014
0.6783913175961018	0.7112344481037169	0.1830407539165719
0.7100202122348236	0.6227402095570440	0.9954104023276216
0.5374029904656581	0.6576060328202189	0.1474505591630972
0.7860534025730743	0.7538551315456559	0.0604010008128029
0.8616938783821553	0.7844100919182324	0.2258427752821959
0.9270092556663414	0.6100948669309147	0.0983317307653451
0.0045707409711966	0.6421822342581081	0.2502769957324631
0.8535312483454405	0.6805947747563650	0.0190093257327177
0.9286992992956939	0.7110831836102847	0.1838509305134295
0.9604665899510110	0.6233382673745638	0.9948501737429112
0.7883415391687336	0.6553554891512334	0.1469415216090297
0.0361963990089293	0.9540438475089132	0.0600102065399568
0.1114035468431496	0.9838677141990217	0.2258259510681597
0.1768359313148906	0.8102666936832179	0.0983425339959254
0.2547988846008238	0.8420730797994125	0.2502445447991306
0.1039471941430676	0.8810329382701099	0.0190196381280288
0.1788929667372727	0.9110803911380785	0.1837912445340706
0.2106502282141918	0.8241313591036104	0.9948669733992119
0.0383341370579160	0.8548866645468323	0.1461978368598125

0.2864093242796741	0.9542790006527042	0.0608039289536748
0.3623080160149001	0.9845962278574234	0.2266699892856536
0.4269149694233576	0.8095344096300110	0.0989941807878907
0.5057432740447041	0.8437590173391456	0.2508151028191321
0.3542879700083503	0.8811974921218635	0.0200307762513177
0.4291789076607131	0.9119896610996179	0.1834614832425672
0.4601968614287344	0.8237802737652373	0.9952924401097079
0.2884451859477508	0.8551046352219132	0.1468237007348279
0.5364962433725964	0.9540071622101678	0.0612207411380297
0.6126127735138454	0.9844386086903967	0.2263657221755793
0.6768678103414629	0.8105760142337672	0.0979580164871976
0.7548902846214602	0.8410805707838236	0.2498618337046281
0.6036346522367069	0.8808923961697275	0.0191400815627034
0.6793507043526503	0.9110697576263433	0.1841425889670987
0.7101569538059138	0.8233667135166840	0.9944044746600653
0.5389669632577423	0.8556544590633735	0.1471369874530595
0.7864964668308332	0.9539203774225768	0.0602566862598906
0.8617203462834411	0.9839622406959165	0.2256065186214897
0.9267627235948694	0.8100120461771922	0.0976971307249244
0.0045120432983126	0.8414332013208394	0.2496728283361733
0.8537450539803046	0.8807665706082091	0.0185362741361885
0.9287760308230659	0.9108546436714349	0.1832374175111795
0.9603597585079413	0.8234626141755328	0.9942238276467279
0.7884033684515188	0.8548455216108977	0.1464384314435847
0.5334587708946898	0.5462207456936596	0.3496823308001211
0.3843634980528421	0.4981404165180479	0.3039392822327400
0.4803128199807816	0.5325086880384333	0.2868873341491565
0.3664351818659028	0.4640587451229621	0.3351174269636843
0.5422199705720558	0.5887932161927409	0.3751359002929249

References

[1] T. Spałek, P. Pietrzyk, Z. Sojka, *J. Chem. Inf. Model.* **2005**, 45, 18.

[2] J. Strunk, M. A. Bañares, I. E. Wachs, *Top. Catal.* **2017**, 60, 1577.

[3] a) A. Amorelli, J. C. Evans, C. C. Rowlands, T. A. Egerton, *J. Chem. Soc., Faraday Trans.* **1987**, 83, 3541; b) S. Neubert, D. Mitoraj, S. A. Shevlin, P. Pulisova, M. Heimann, Y. Du, G. K. L. Goh, M. Pacia, K. Kruczala, S. Turner, W. Macyk, Z. X. Guo, R. K. Hocking, R. Beranek, *J. Mater. Chem. A* **2016**, 4, 3127; c) J. Patzsch, Jacob N. Spencer, A. Folli, J. Z. Bloh, *RSC Adv.* **2018**, 8, 27674.

[4] M. Jabłońska, G. Delahay, K. Kruczała, A. Błachowski, K. A. Tarach, K. Brylewska, C. Petitto, K. Góra-Marek, *J. Phys. Chem. C* **2016**, 120, 16831.

[5] S. Yu, H. J. Yun, D. M. Lee, J. Yi, *J. Mater. Chem.* **2012**, 22, 12629.

[6] K. Gołąbek, A. E. Palomares, J. Martínez-Triguero, K. A. Tarach, K. Kruczała, V. Girman, K. Góra-Marek, *Appl. Catal. B: Environ.* **2019**, 259, 118022.

[7] B. V. Kerr, H. J. King, C. F. Garibello, P. R. Dissanayake, A. N. Simonov, B. Johannessen, D. S. Eldridge, R. K. Hocking, *Energy Fuels* **2022**, 36, 2369.

[8] a) B. Kortewille, O. Pfingsten, G. Bacher, J. Strunk, *ChemPhotoChem* **2022**, 6, e202100120; b)

B. Kortewille, I. E. Wachs, N. Cibura, O. Pfingsten, G. Bacher, M. Muhler, J. Strunk, *ChemCatChem* **2018**, 10, 2360; c) B. Kortewille, I. E. Wachs, N. Cibura, O. Pfingsten, G. Bacher, M. Muhler, J. Strunk, *Eur. J. Inorg. Chem.* **2018**, 2018, 3725.

[9] a) Ł. Łańcucki, S. Schlick, M. Danilczuk, F. D. Coms, K. Kruczała, *Polym. Degrad. Stab.* **2013**, 98, 3; b) E. Finkelstein, G. M. Rosen, E. J. Rauckman, *Arch. Biochem. Biophys.* **1980**, 200, 1.

[10] E. Finkelstein, G. M. Rosen, E. J. Rauckman, J. Paxton, *Mol. Pharmacol.* **1979**, 16, 676.

[11] D. K. Pallotti, L. Passoni, P. Maddalena, F. Di Fonzo, S. Lettieri, *J. Phys. Chem. C* **2017**, 121, 9011.

[12] A. Zaban, M. Greenshtein, J. Bisquert, *ChemPhysChem* **2003**, 4, 859.

[13] C. Adler, D. Mitoraj, I. Krivtsov, R. Beranek, *J. Chem. Phys.* **2020**, 152, 244702.

[14] a) G. Kresse, J. Furthmüller, *Phys. Rev. B* **1996**, 54, 11169; b) G. Kresse, J. Furthmüller, *Comp. Mater. Sci.* **1996**, 6, 15; c) G. Kresse, D. Joubert, *Phys. Rev. B* **1999**, 59, 1758.

[15] a) P. Hohenberg, W. Kohn, *Phys. Rev.* **1964**, 136, B864; b) W. Kohn, L. J. Sham, *Phys. Rev.* **1965**, 140, A1133.

[16] J. P. Perdew, K. Burke, M. Ernzerhof, *Phys. Rev. Lett.* **1996**, 77, 3865.

[17] M. Cococcioni, S. de Gironcoli, *Phys. Rev. B* **2005**, 71, 035105.

[18] S. L. Dudarev, G. A. Botton, S. Y. Savrasov, C. J. Humphreys, A. P. Sutton, *Phys. Rev. B* **1998**, 57, 1505.

[19] E. Finazzi, C. Di Valentin, G. Pacchioni, A. Selloni, *J. Chem. Phys.* **2008**, 129.

[20] S. Lutfalla, V. Shapovalov, A. T. Bell, *J. Chem. Theory Comput.* **2011**, 7, 2218.

[21] N. J. Mosey, P. Liao, E. A. Carter, *J. Chem. Phys.* **2008**, 129.
